# Inside-outward breakup of sea ice by locally generated waves in the Vincennes Bay Polynya

**DOI:** 10.1038/s41467-026-77979-y

**Published:** 2026-09-29

**Authors:** Joey J. Voermans, Qingxiang Liu, Lang Cao, Josh Kousal, Petra Heil, Alexander D. Fraser, Clarence O. Collins, Jean Rabault, Alexander V. Babanin

**Affiliations:** 1 Department of Infrastructure Engineering, University of Melbourne, Melbourne, VIC Australia; 2 State Key Laboratory of Physical Oceanography, Ocean University of China, Qingdao, China; 3 European Centre for Medium-Range Weather Forecasts (ECMWF), Bonn, Germany; 4 British Antarctic Survey, Cambridge, United Kingdom; 5 Australian Antarctic Program Partnership, Institute for Marine and Antarctic Studies, University of Tasmania, nipaluna/Hobart, TAS Australia; 6 Institute for Marine and Antarctic Studies, University of Tasmania, nipaluna/Hobart, TAS Australia; 7 U.S. Army Engineer Research and Development Center, Coastal and Hydraulics Laboratory, Field Research Facility, Duck, NC USA; 8 Norwegian Meteorological Institute, Oslo, Norway

**Keywords:** Physical oceanography, Cryospheric science

## Abstract

Coastal polynyas are recurring regions of open water within sea ice that serve as sea-ice factories, producing vast amounts of new ice relative to nearby sea-ice-covered regions. Their formation and persistence are commonly attributed to strong offshore winds, while the influence of waves on polynya dynamics remains largely unexplored. Using rare wave-buoy observations from the Vincennes Bay Polynya, we show that strong winds generate energetic waves capable of breaking surrounding sea ice from the inside-outward, contrasting with outside-inward breakup caused by Southern Ocean swells. Satellite records from 2003–2022 reveal that extreme wave-induced breakup events coincide with substantial reductions in sea-ice concentration that persist for at least one week, accompanied by increased transitions to active frazil ice. These findings demonstrate that locally generated waves impact polynya dynamics and reshape surrounding sea-ice conditions. As Antarctic coastal polynyas evolve under a changing climate, wave activity may become increasingly important in shaping their sea-ice conditions and evolution.

## Abstract (web summary)

Wave-buoy and satellite data from an Antarctic polynya show that wind-driven waves can break surrounding sea ice from the inside out, with extreme events coinciding with persistent sea-ice changes, revealing a new role for waves in polynya dynamics.

## Introduction

Polynyas are persistent regions of open water, or very thin ice, within the sea-ice cover. They are formed and maintained either thermally, where heat prevents new ice formation and may thin or melt existing sea ice, or mechanically, through wind or current-driven advection and divergence of sea ice^1^. They play a crucial role in polar climate by enhancing ocean-atmosphere heat and moisture exchange, increasing solar radiation absorption, promoting biological productivity^2^, and providing areas for the generation of sea ice^3,4^. The associated brine rejection increases upper-ocean salinity and can contribute to dense shelf water formation^5^. Understanding the processes that form, maintain, and modify polynyas is therefore important for assessing their role in polar climate.

Coastal polynya behavior is commonly understood in terms of atmospheric forcing and coastal geometry, including wind, air temperature, and the distribution of landfast ice and grounded icebergs, which are widely recognized as key controls on polynya formation and persistence^6–8^. Yet waves are rarely considered in this context, even though polynyas can provide favorable conditions for the generation of energetic wave fields. Strong katabatic winds and open-water fetches, often tens of kilometers in scale, allow for substantial wave growth. For example, Ackley et al.^9^ observed significant wave heights exceeding 2 m in Terra Nova Bay during 20 m/s winds, and Herman & Bradtke^10^ identified similarly developed wave fields from satellite imagery. These observations suggest that energetic waves are not exceptional in coastal polynyas, but may be a recurring part of the polynya environment, capable of influencing sea-ice drift, rafting, frazil-ice herding, and sea-ice breakup^5,11,12^.

Despite this evidence of energetic wave activity, the characteristics and influence of these waves within Antarctic coastal polynyas and the surrounding sea ice remain largely unknown because in situ observations are exceptionally scarce. Beyond a few documented cases^9,10,13^, sustained measurements remain virtually absent. This scarcity reflects the severe logistical challenges of deploying instruments during extreme wind events, as such hazardous and unpredictable conditions are typically avoided by research expeditions. Even when instruments are successfully deployed, they may drift rapidly across the open water into adjacent consolidated ice, further limiting the duration of observations. While upward-looking acoustic Doppler current profilers provide a viable alternative for measuring waves from beneath the surface^14,15^, deploying such moored instrumentation in Antarctic coastal polynyas is logistically demanding and resource-intensive. Consequently, acquiring sustained in situ records of wave activity in polynyas remains a major experimental challenge.

**Fig. 1:**
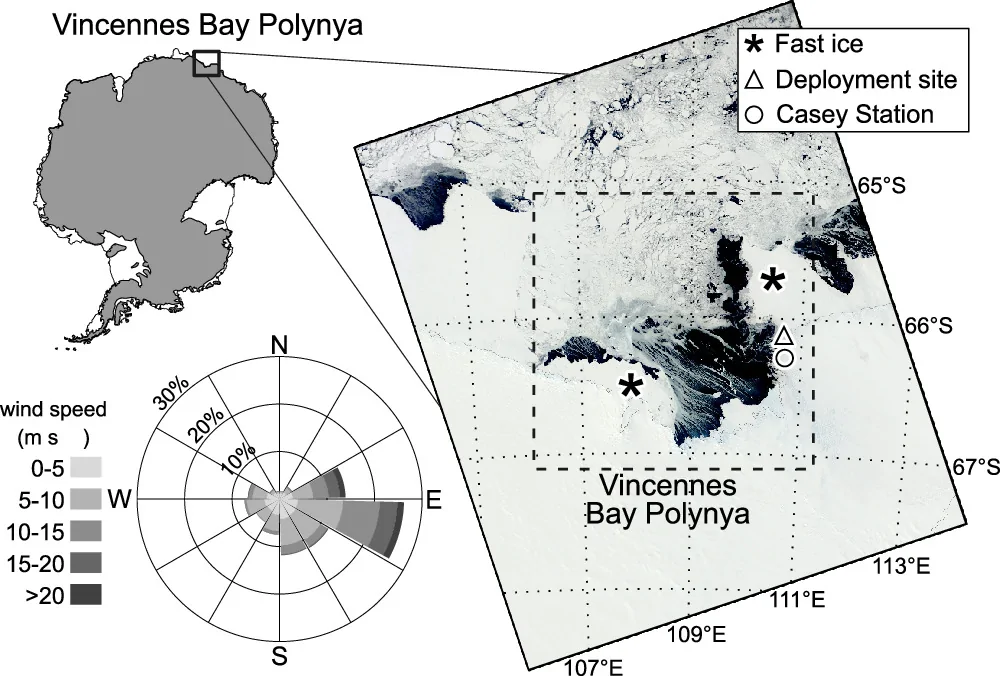
Overview of the Vincennes Bay Polynya study region. The MODIS image, accessed via NASA Worldview, shows a representative polynya opening on 9 November 2023, with the deployment site, Casey Station and the northward-extending fast ice tongue indicated. The wind rose shows ERA5 wind conditions during August–October 2003-2022 at 65.5^∘^S, 108.5^∘^E.

**Fig. 2:**
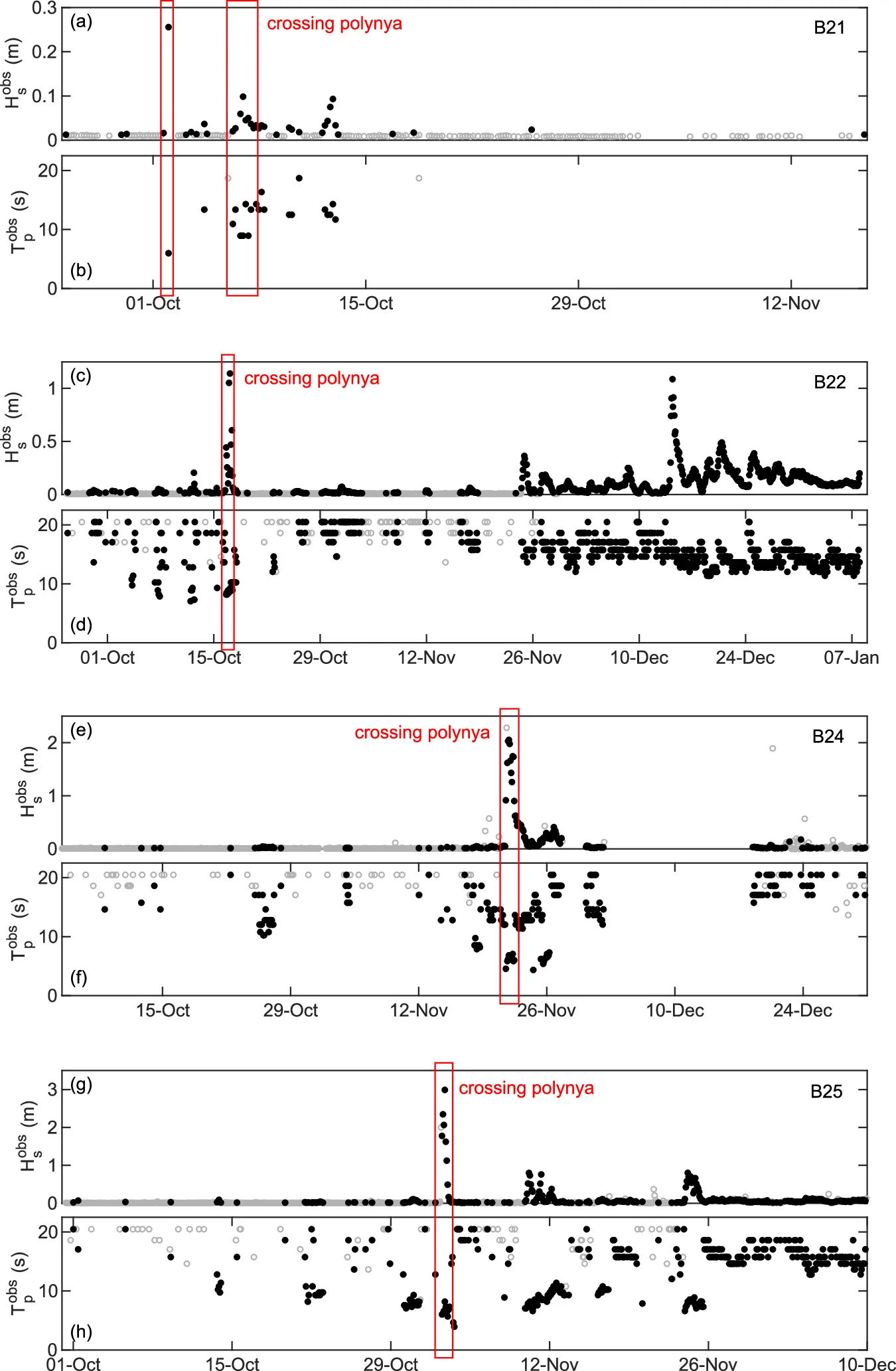
Wave observations during the buoy deployments. Buoy measurements of significant wave height ${H}_{s}^{{{{\rm{obs}}}}}$ and peak wave period ${T}_{p}^{{{{\rm{obs}}}}}$ for B21 (**a**, **b**), B22 (**c**, **d**), B24 (**e**, **f**), and B25 (**g**, **h**). The buoy crossings of the polynya are highlighted by the red boxes. Gray markers indicate observations affected by the instrumental noise floor.

Over recent years, we deployed a small number of sea-ice buoys [see “Methods” ^16,17^], on landfast sea ice (fast ice) near Casey Station, East Antarctica (Fig. 1), to investigate wave-ice interactions^18,19^. The deployment region borders the Vincennes Bay Polynya, a medium-class coastal polynya that produces substantial volumes of sea ice^6^ and dense shelf water formation linked to Antarctic Bottom Water^20,21^. Most buoys were recovered and redeployed in subsequent seasons, but on several occasions the fast ice on which they were installed broke out, causing the buoys to drift freely across the polynya. During these events, the instruments recorded a sharp increase in wave energy immediately following breakout (Fig. 2a, c, e, g) with relatively short dominant wavelengths (Fig. 2b,d,f,h), implying that the observed wave field was dominated by locally generated waves rather than the long-period Southern Ocean swell typical of the Antarctic pack ice. Such young, steep waves may therefore drive sea-ice break-up from within the polynya and thereby modify the surrounding ice cover.

Here, we combine in situ buoy and satellite observations to examine locally generated waves in the Vincennes Bay Polynya and their influence on the surrounding sea ice. We demonstrate that the observed wave conditions are consistent with fetch-limited growth and assess associated changes in sea-ice properties within and around the polynya. Our results indicate that locally generated waves can be sufficiently steep to fracture sea ice from the inside-out and modify the surrounding sea-ice cover. This reveals a largely overlooked mechanism through which waves influence sea-ice conditions in the Vincennes Bay Polynya and, more broadly, the dynamics of Antarctic coastal polynyas.

## Results

### Swell or wind waves?

We use measurements from four individual drifting wave–ice buoys to characterize the wave field within the Vincennes Bay Polynya during and shortly after the fast ice breakout, particularly to assess whether the waves were generated locally or represent distant swell. The buoys, hereafter B21, B22, B24 and B25, were deployed on fast ice near Casey Station during the 2021, 2022, 2024 and 2025 field campaigns, respectively (see “Methods”).

**Fig. 3:**
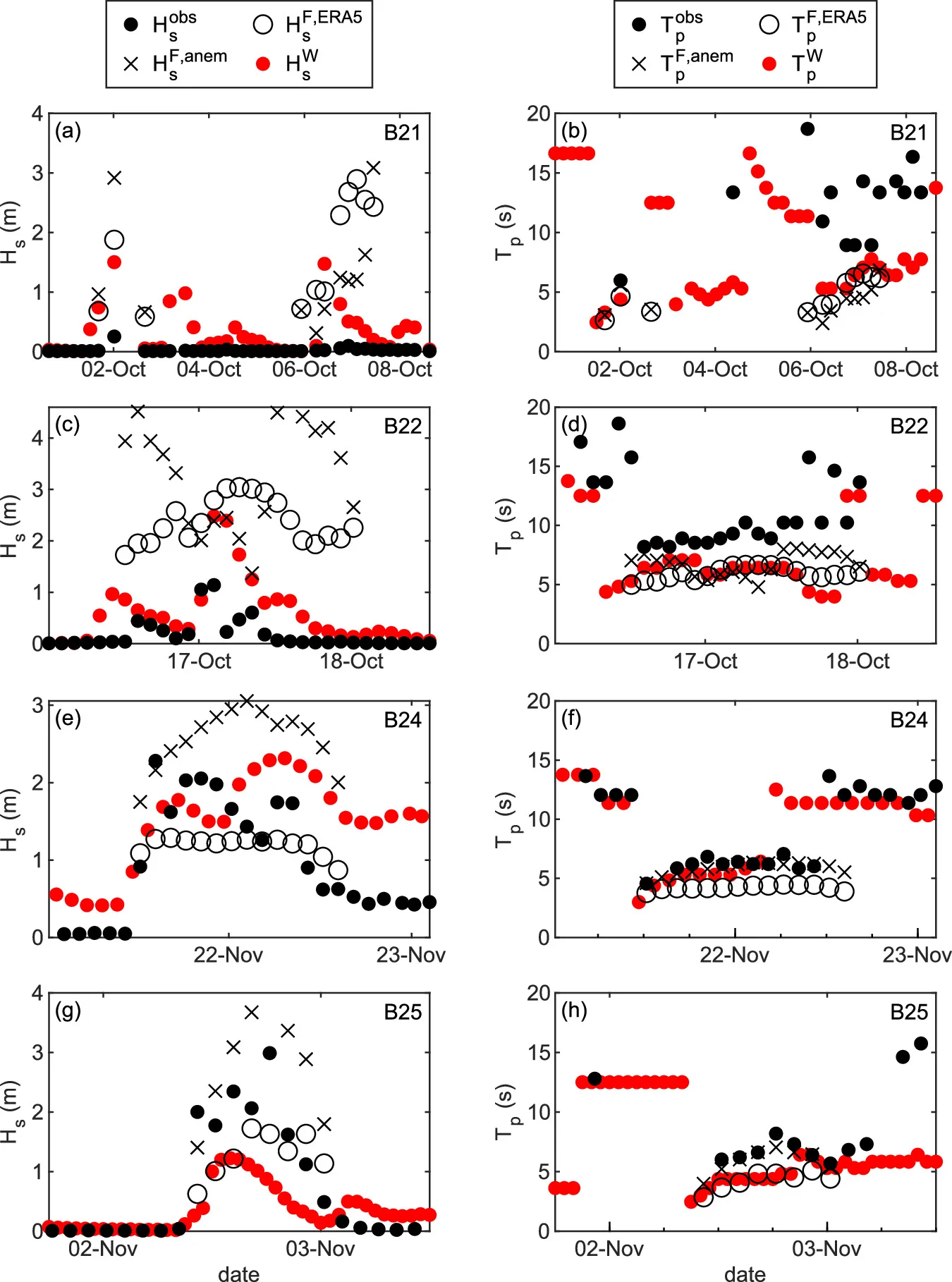
Observed and expected wave conditions during polynya crossings. Comparison between the buoy observations of significant wave height *H*_*s*_ and peak wave period *T*_*p*_ during the polynya crossing and expected wave conditions following fetch-limited wave theory for B21 (**a**, **b**), B22 (**c**, **d**), B24 (**e**, **f**) and B25 (**g**, **h**). Solid black circles represent the buoy observations, crosses and open circles represent estimates using anemometer and ERA5 winds, respectively, following the fetch-limited wave growth relations. Red solid circles are WWIII simulations. Superscripts `obs', `F' and `W' denote observed values, estimates from fetch-limited growth relations, and WWIII simulations, respectively. Subscripts `ERA5', `anem' and `anem, buoy' indicate winds from ERA5 reanalysis, Casey Station anemometer observations, and buoy measurements, respectively.

A common indicator for distinguishing wind waves from swell is the empirical wave-age criterion *c*_*p*_/*U*_10_ < 1.2, which is satisfied while the buoys are crossing the polynya (Suppl. Fig. S1), indicating a wind-wave regime. However, because sufficiently strong winds can also produce low wave ages in the presence of swell, we further characterize the buoy observations within their broader wind, ice and wave environment using WAVEWATCH III simulations^22^,(hereafter WWIII, see “Methods”). The WWIII simulations reproduce the observed bulk wave characteristics, including significant wave height ${H}_{s}^{{{{\rm{W}}}}}$ and peak period ${T}_{p}^{{{{\rm{W}}}}}$ (Fig. 3), reasonably well for B24, given the inherent uncertainty in the wind and ice input fields. For B25, WWIII underestimates both peak period and significant wave height. As shown in the following section, this discrepancy is well explained by an approximately twofold underestimation of wind speed in ERA5 (Suppl. Fig. S1d). In contrast, for B21 and B22, WWIII underestimates the observed peak periods and overestimates the significant wave heights. This mismatch likely reflects wave attenuation by sea ice surrounding the buoys near the polynya edge (Suppl. Figs. S2–S9), which reduced the wave energy measured by the instruments. Although WWIII includes parameterized wave attenuation by sea ice, reproducing the exact magnitude of this damping would require detailed information on ice concentration, ice thickness and floe size, as well as higher-fidelity wind forcing, which are not available here^10,23^. Further tuning of the model source terms could improve the comparison, but such refinements are beyond the scope of this study.

**Fig. 4:**
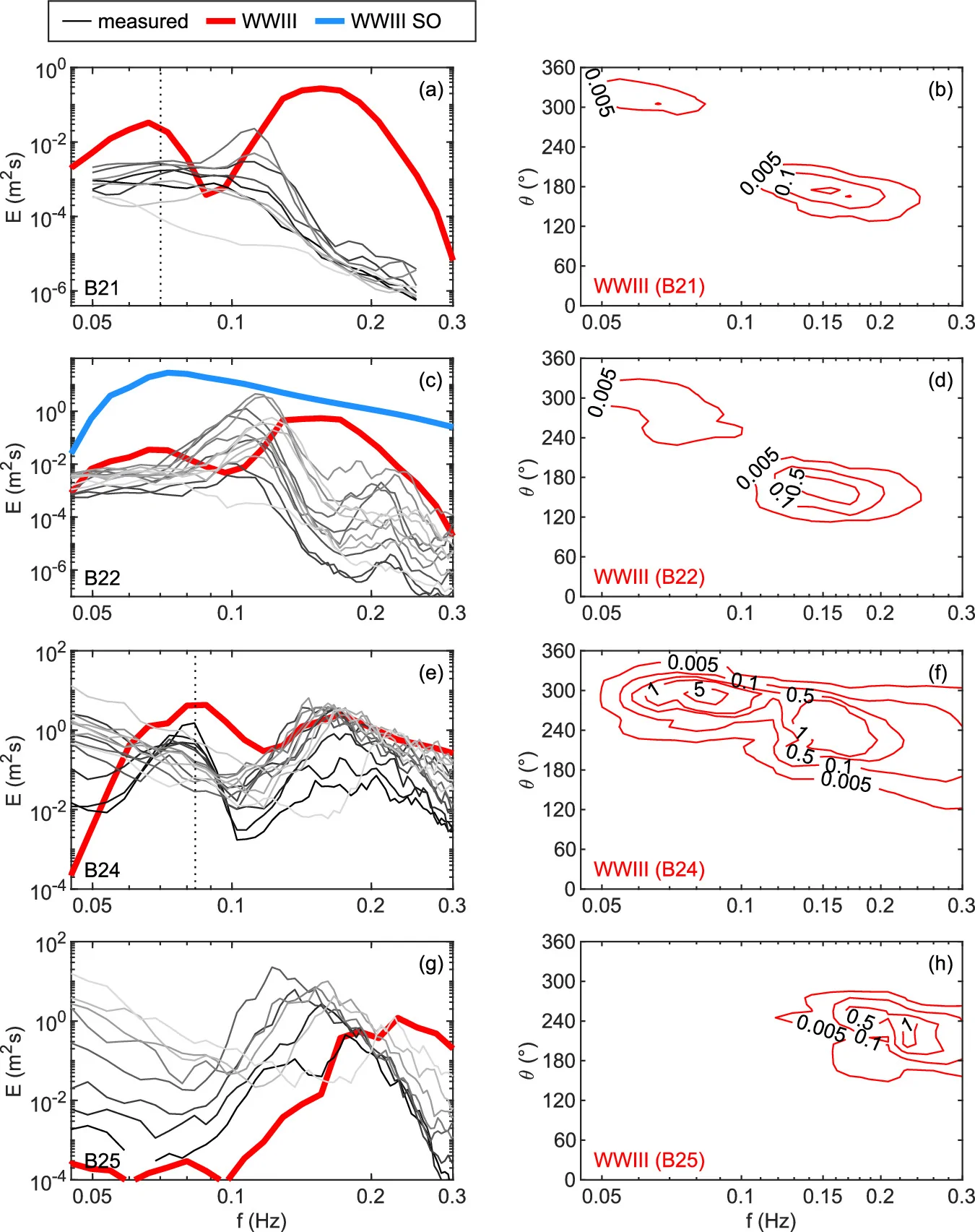
Wave spectra during polynya crossings. Gray-scale shading from light to dark denotes the progression in time, with lighter tones representing spectra measured earlier and darker tones later surrounding the polynya crossing periods for (**a**) B21 on 6–7 October 2021, (**c**) B22 on 17–18 October 2022, (**e**) B24 on 21–22 November 2024, and (**g**) B25 on 2 November 2025. Representative WWIII spectra at the buoy locations are shown in red; panel (**b**) additionally shows the simulated spectrum near the Southern Ocean ice edge. Panels (**b**, **d**, **f**, **h**) show the WWIII directional spectra corresponding to the frequency spectra in (**a**, **c**, **e**, **g**), respectively. Vertical dotted lines in (**a**, **e**) indicate peak wavelengths estimated from SAR observations. The instrumental noise floor scales approximately as *f*^−4^, with *E* ≈ 4 × 10^−5^ m^2^s at *f* = 0.1 Hz^17^.

The spectral composition of the buoy measurements is compared with the simulated WWIII directional spectra to distinguish the evolution of locally generated wind waves from the possible contribution of Southern Ocean swell. With the exception of B25, the simulated WWIII directional spectra contain two distinct peaks: one associated with locally generated waves within the polynya and another with swell propagating from the Southern Ocean, as indicated by their respective directions (Fig. 4b, d, f). For B24, the simulated swell peak period agrees with both the measured peak at the buoy location and SAR-derived observations in the Antarctic pack ice (Suppl. Fig. S10), supporting the interpretation that this observed component represents Southern Ocean swell. In the buoy observations, however, the swell component is strongly attenuated, being barely detectable for B21 and B22 and clearly discernible only for B24. This indicates that most swell energy was dissipated by the several hundred kilometers of pack ice separating the buoys from the Southern Ocean ice edge, and that WWIII overestimates the amount of swell energy reaching the polynya. Thus, while Southern Ocean swell may be present as a weak low-frequency component, the buoy-measured wave energy is dominated by locally generated wind waves.

The individual records provide further context for this interpretation. For B21, spectral energy at *f* > 0.16 Hz (*T* < 6 s) approaches the instrument noise floor, indicating strong attenuation of higher-frequency waves by the sea ice surrounding the buoy. For B22, the gradual shift of the dominant spectral peak toward lower frequencies is consistent with the growth of locally generated waves under increasing fetch or strengthening winds, rather than the arrival of Southern Ocean swell. This interpretation is supported by the 150–200 km of sea ice separating the polynya from the open-ocean ice edge. SAR observations from 18 October 2022 at 12:38 UTC showed 11–12 s swell with *H*_*s*_ = 1–1.5 m immediately north of the ice edge (XWaves, Ifremer), whereas no corresponding long-period energy above the buoy noise floor was detected within the polynya at B22 (Fig. 4c). The absence of this swell signal indicates that it was effectively attenuated before reaching the buoy, supporting the interpretation that the observed spectral peak was associated with locally generated wind waves. The origin of the secondary, higher-frequency peak near *f* = 0.2 Hz remains uncertain without directional measurements, but it may reflect nonlinear transfer of energy from the dominant peak to its doubled-frequency harmonic through wave–wave triad interactions^24^. For B25, the spectral mismatch is primarily attributable to underestimated ERA5 wind forcing, while the steepening of the observed high-frequency tail suggests an additional influence of local sea ice (Fig. 4g).

Overall, our analysis indicates that the wave energy measured within the Vincennes Bay Polynya during breakout was dominated by locally generated waves, while Southern Ocean swell was strongly attenuated across the intervening pack ice and contributed little to the buoy-measured wave energy.

### Fetch-limited wave growth

We next assess whether the observed locally generated waves developed according to established fetch-limited growth relationships for open-water conditions. Using standard fetch-limited growth equations (see Methods), we estimate the expected significant wave height (${H}_{s}^{{{{\rm{F}}}}}$) and peak period (${T}_{p}^{{{{\rm{F}}}}}$) (Fig. 3) using wind speeds from ERA5 reanalysis and the land-based anemometer at Casey Station. For B24 and B25, the observed peak periods are closely reproduced when the Casey anemometer winds are used, while the observed significant wave heights generally fall between the estimates obtained from the ERA5 and Casey wind speeds. Because the fetch-limited estimates do not include wave attenuation by sea ice, ${H}_{s}^{{{{\rm{F}}}}}$ is generally overestimated for B21 and B22 (Fig. 3a, c). This is consistent with attenuation by sea ice between the buoys and the polynya ice edge.

**Fig. 5:**
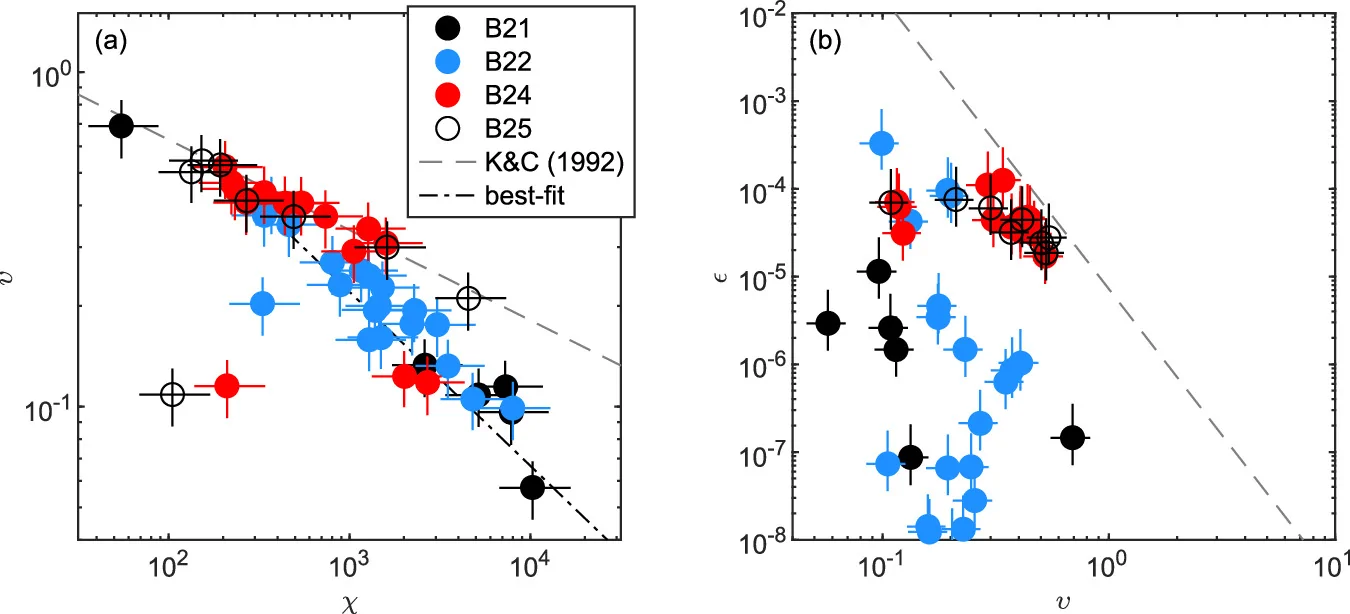
Fetch-limited wave growth during polynya crossings. Comparison of the (**a**) non-dimensional wave fetch $\chi=gF/{\left({U}_{10}^{{{{\rm{anem}}}}}\right)}^{2}$ and (**b**) wave energy $\epsilon=\frac{1}{16}{g}^{2}{\left({H}_{s}^{F}\right)}^{2}/{\left({U}_{10}^{{{{\rm{anem}}}}}\right)}^{4}$ against peak frequency $\upsilon={f}_{p}^{F}{U}_{10}^{{{{\rm{anem}}}}}/g$ for the data presented in Fig. 3. We excluded the first three data points of B21 at the second polynya crossing. The dashed line corresponds to the relation of^25^, and a best fit to the data in (**a**) is given by the dashed-dot-line, with *υ* ∝ *χ*^−0.52^.

In Fig. 5a, the observed non-dimensional peak frequency (*υ*) and fetch (*χ*) are compared with the open-water fetch-limited growth relation of^25^ (see “Methods”). While the observations from B24 and B25 follow this relation well, B22 exhibits a systematically different trend, scaling approximately as *υ* ∝ *χ*^−0.52^. In other words, the peak period appears to increase at a considerably higher rate than expected for open-water conditions under comparable wind and fetch. Uncertainties in wind speed (*U*_10_) and fetch (*F*) cannot account for this deviation, even when a 20% uncertainty is assumed for both parameters this trend persists (Fig. 5a). Similarly, the non-dimensional energy from B24 and B25 aligns well with the fetch-limited wave growth relation (Fig. 5b), whereas the energy measured by B22 is substantially lower.

**Fig. 6:**
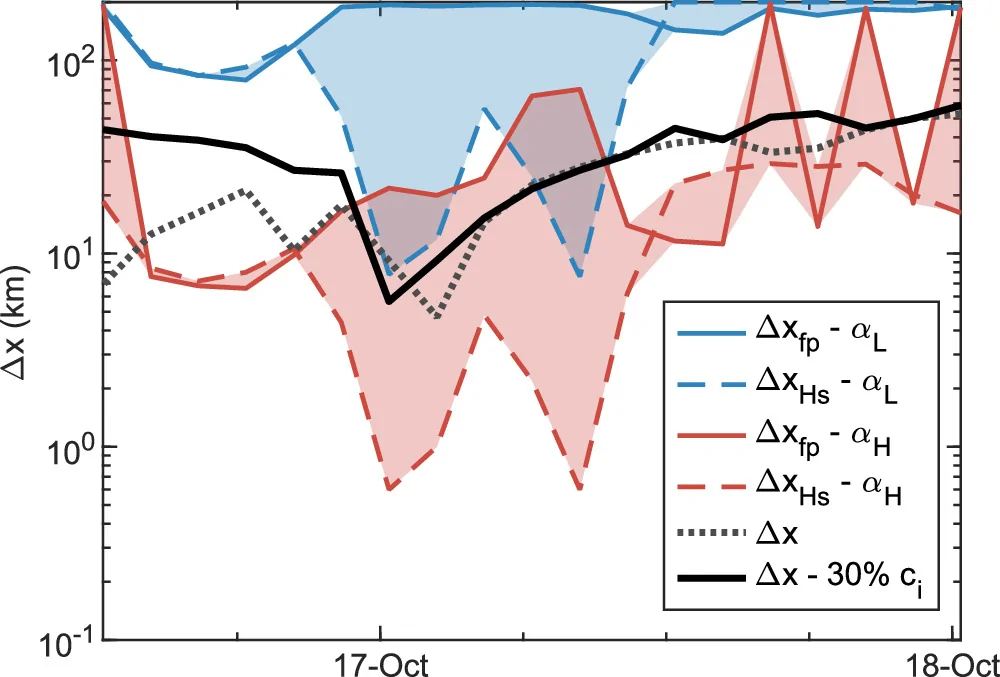
Wave propagation distances through sea ice. Comparison of the estimated distance from the downwind polynya ice edge to the buoy, *Δx*, with the effective propagation distances through sea ice required to reproduce the buoy-observed significant wave height and peak frequency for B22, *Δx*_*Hs*_ and *Δx*_*fp*_, respectively. Estimates of *Δx*_*Hs*_ and *Δx*_*fp*_ are shown for a low attenuation rate (*α*_*L*_), representative of pancake ice, and a high attenuation rate (*α*_*H*_), representative of fast ice. The dashed black line denotes the distance from the buoy to the polynya ice edge (i.e., defined by the 60% sea-ice-concentration contour following^27^), while the solid black line denotes the distance from the buoy to the 30% sea-ice-concentration contour. Blue and red shading indicate the separation between *Δx*_*Hs*_ and *Δx*_*fp*_ under the low- and high-attenuation scenarios, respectively.

To show that wave attenuation by sea ice can explain the anomalous wave growth observed for B22, we first construct a JONSWAP spectrum^26^ using the significant wave height ${H}_{s}^{F}$ and peak frequency ${f}_{p}^{F}$ predicted from the open-water fetch-limited growth relations for the local wind and fetch conditions. We then apply two sea-ice attenuation regimes to the spectrum: a low rate (Eq. (3)) representative of pancake ice, and a high rate (*α*_*H*_ = 1.3 × 10^−1^*f*^2.66^) representative of fast ice^19^. We then determine the effective distance $\Delta {x}_{{f}_{p}}$ required for the attenuated spectral peak to reproduce ${f}_{p}^{{{{\rm{obs}}}}}$ and, equivalently, the effective distance $\Delta {x}_{{H}_{s}}$ required for the attenuated spectrum to reproduce ${H}_{s}^{{{{\rm{obs}}}}}$. If these independently inferred distances are comparable, the observed reduction in wave energy and peak frequency can be explained by attenuation by sea-ice rather than by a departure from fetch-limited wave growth. Using this approach, we find that $\Delta {x}_{{H}_{s}}$ and $\Delta {x}_{{f}_{p}}$ converge for times before 17 October 2022 and after 12:00 on 17 October 2022 (Fig. 6). This convergence, together with the satellite-derived *Δx* falling within the range predicted by the two attenuation scenarios, indicates that the deviations from fetch-limited growth can be readily explained by sea-ice attenuation. In contrast, during the early hours of 17 October 2022, $\Delta {x}_{{H}_{s}}$ and $\Delta {x}_{{f}_{p}}$ diverge substantially, suggesting that attenuation alone cannot account for the observations. The strong drop in wind speed recorded by the land-based anemometer around this time likely played a role.

### Wave-induced break-up

To assess the broader influence of wind waves on sea ice within and around the Vincennes Bay Polynya, we use the wave-induced ice break-up parameter (*I*_br_)^18^. This parameter links the wave field to the mechanical response of the sea-ice cover and identifies conditions under which the waves are sufficiently energetic to fracture consolidated ice. Since the observed waves within the polynya follow the fetch-limited growth relations well, we use the open-water relations of^25^ to estimate the significant wave height (${H}_{s}^{{{{\rm{F}}}}}$) and corresponding peak wavelength (${\lambda }_{p}^{{{{\rm{F}}}}}$). These wave parameters are derived from daily-averaged ERA5 wind speed and direction and the long-term polynya-edge climatology of^27^, which together provide the effective daily fetch. From these, we calculate *I*_br_ to identify periods when locally generated waves could induce sea-ice break-up and assess the associated evolution of the sea-ice cover.

**Fig. 7:**
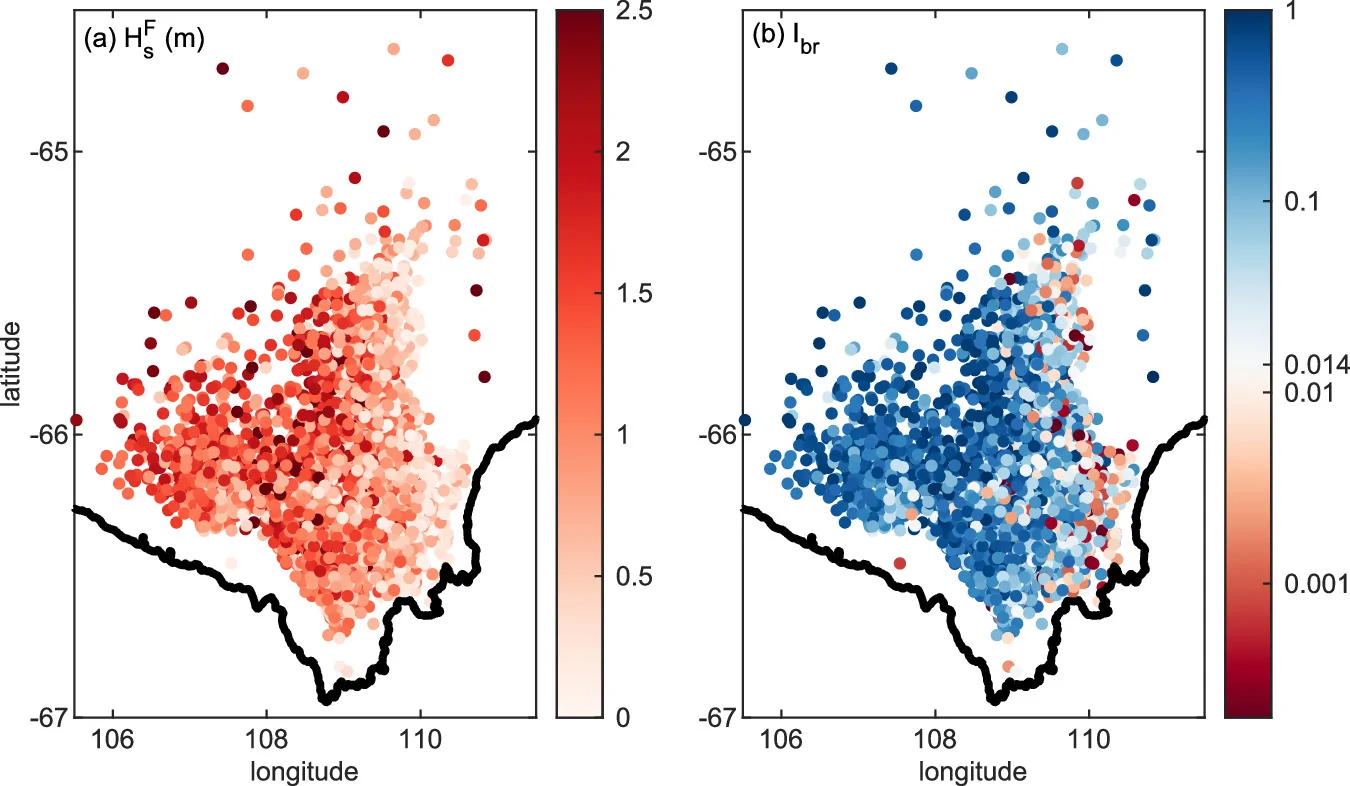
Spatial distribution of extreme wave conditions and break-up potential. The largest expected daily-averaged wave height (**a**) based on fetch-limited conditions and corresponding break-up parameter *I*_*br*_ (**b**) are shown for the period 2003–2022 (April–October). We jitter the longitude and latitude positions of these coordinates in the figure to avoid overlap along a fixed grid due to spatial resolution of the datasets, using a normal distribution with a mean of zero and standard deviation of 0.02^∘^.

Figure 7 a shows the spatial distribution of the estimated ${H}_{s}^{{{{\rm{F}}}}}$, where each point marks the location of the largest expected significant wave height in the polynya for a given day, corresponding to the downwind position along the polynya boundary with the longest fetch. The maximum daily ${H}_{s}^{{{{\rm{F}}}}}$ in the Vincennes Bay Polynya has a median of 1.07 m and a 95th percentile of 2.02 m, corresponding to *T*_*p*_ values of 4.1 and 5.6 s, respectively. The largest values occur along the north-western boundary of the polynya, consistent with the prevailing wind direction in this region (Fig. 1). The corresponding spatial distribution of the wave-induced break-up parameter for an ice thickness of *h* = 0.5 m is shown in Fig. 7b, where blue shading indicates conditions exceeding the critical threshold for fracture (*I*_br_ > 0.014). While uncertainties remain in the parameters used to evaluate *I*_br_, including assumptions about the (daily averaged) wind forcing and sea ice mechanical strength, 64% of all cases exceed the threshold by at least one order of magnitude. For thin ice at the polynya edge, represented by *h* = 0.16 m^28^, this proportion increases to 90%. These results indicate that waves generated within the polynya are generally energetic enough to fracture the sea ice at the polynya boundary, at least for floes with sizes comparable to or larger than the peak wavelength. Consequently, the floe-size distribution near the polynya margin, and therefore the local sea ice conditions, are likely influenced by the prevailing wave field within the polynya.

To examine whether extreme wave events also affect the broader sea ice cover in and around the polynya, we analyze satellite-derived changes in sea ice concentration using a conditional averaging approach applied to AMSR-E and AMSR2 observations from 2003-2022, focusing on events of extreme *I*_br_ (see “Methods”). To minimize the influence of the seasonal advance and retreat of the ice edge, the analysis is restricted to August–October, when the polynya is fully developed and its extent is less affected by background ice growth. Notably, the breakout days of B21 and B22 both fall within the extreme-*I*_br_ subset, indicating that the in situ observations sampled the high-break-up-potential conditions targeted by the broader statistical analysis.

**Fig. 8:**
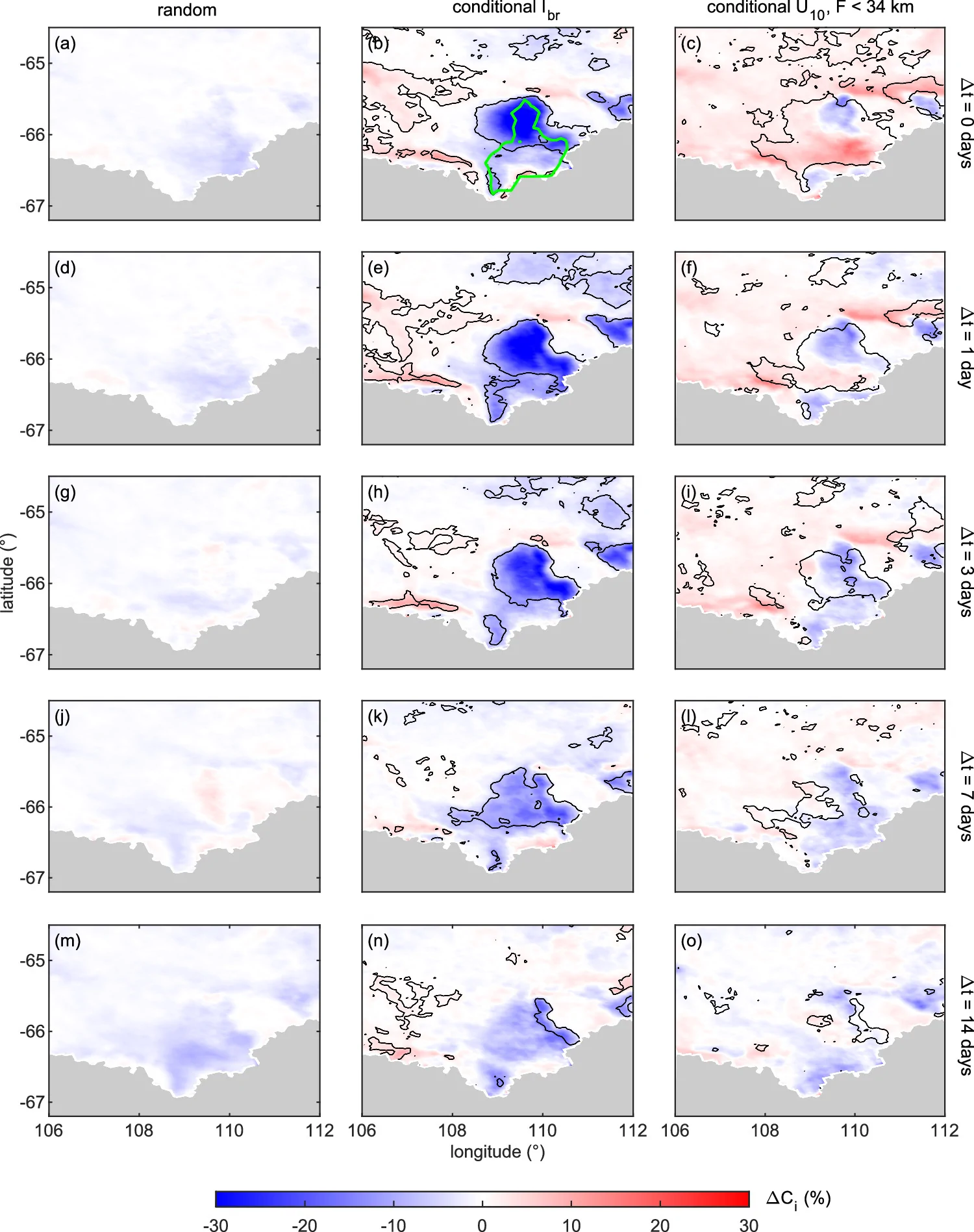
Sea-ice concentration changes following extreme events. Conditionally-averaged sea ice concentration difference maps are shown for August–October, relative to three days before the samples. First column is based on random sampling (**a**, **d**, **g**, **j**, **m**), second column is sampled based on extremes in *I*_*br*_ (**b**, **e**, **h**, **k**, **n**), and the third column is sampled based on extreme wind events with short fetches (**c**, **f**, **i**, **l**, **o**). Rows represent the lag *Δt* relative to the extreme event. Black contours in the second column identify *p* < 0.05 relative to the random subset, and in the third column relative to the extreme-*I*_*br*_ subset. The green contour in (**b**) identifies the region at which the polynya was present for at least 10% of the subset.

Figure 8 presents the mean change in sea ice concentration (*Δc*_*i*_) relative to conditions prior to the extreme event, shown for *Δt* = 0, 1, 3, 7, and 14 days following the days of extreme *I*_br_ during August–October. The left column shows the randomly selected subset, while the middle column shows the conditionally sampled subset based on extreme *I*_br_ values. Regions enclosed by black contours in the middle column denote areas where the conditional and random sets differ significantly (*p* < 0.05). A representative polynya edge, shown in green in Fig. 8b, corresponds to the contour where the polynya was present at least 10% of the time across the subset.

Extreme *I*_br_ events are associated with a pronounced and statistically significant decrease in sea ice concentration within the Vincennes Bay Polynya and its immediate surroundings, persisting for at least seven days, with reductions of up to about 30%. Although differences relative to the random subset remain visible beyond this period, they are no longer statistically significant and gradually return toward the random subset conditions (*p* > 0.05; Fig. 8n). The pronounced decrease in sea-ice concentration within and immediately around the polynya is accompanied by a distinct and statistically significant increase farther downwind.

As extreme *I*_br_ events are necessarily associated with strong winds, we next test whether the observed sea-ice response can be explained by wind forcing alone. We therefore examine sea-ice concentration anomalies during extreme wind events with short fetches (*F* < 34 km; see “Methods”), for which the potential for fetch-limited wave growth is strongly reduced. Regions enclosed by black contours in the right column of Fig. 8 indicate areas where the *I*_br_- and short-fetch *U*_10_-conditioned subsets differ significantly (*p* < 0.05). In contrast to the broad reduction in sea ice concentration under the *I*_br_ criterion, the short-fetch wind subset shows no significant polynya-scale decrease. Instead it exhibits distinct positive anomalies around and downwind of the polynya, with observed differences against the *I*_br_ subset remaining statistically significant up to *Δt* = 3 days. These results demonstrate that wave forcing has an additional and measurable impact on sea ice reduction beyond that associated with wind forcing alone.

A similar conditional analysis can be performed for sea-ice thickness using SMOS, although interpretation is complicated by the thin-ice limitation and the results should be treated with caution. In general, reductions in sea-ice concentration are accompanied by corresponding decreases in thickness within and around the polynya, offset by increases farther afield (Suppl. Fig. S11).

**Fig. 9:**
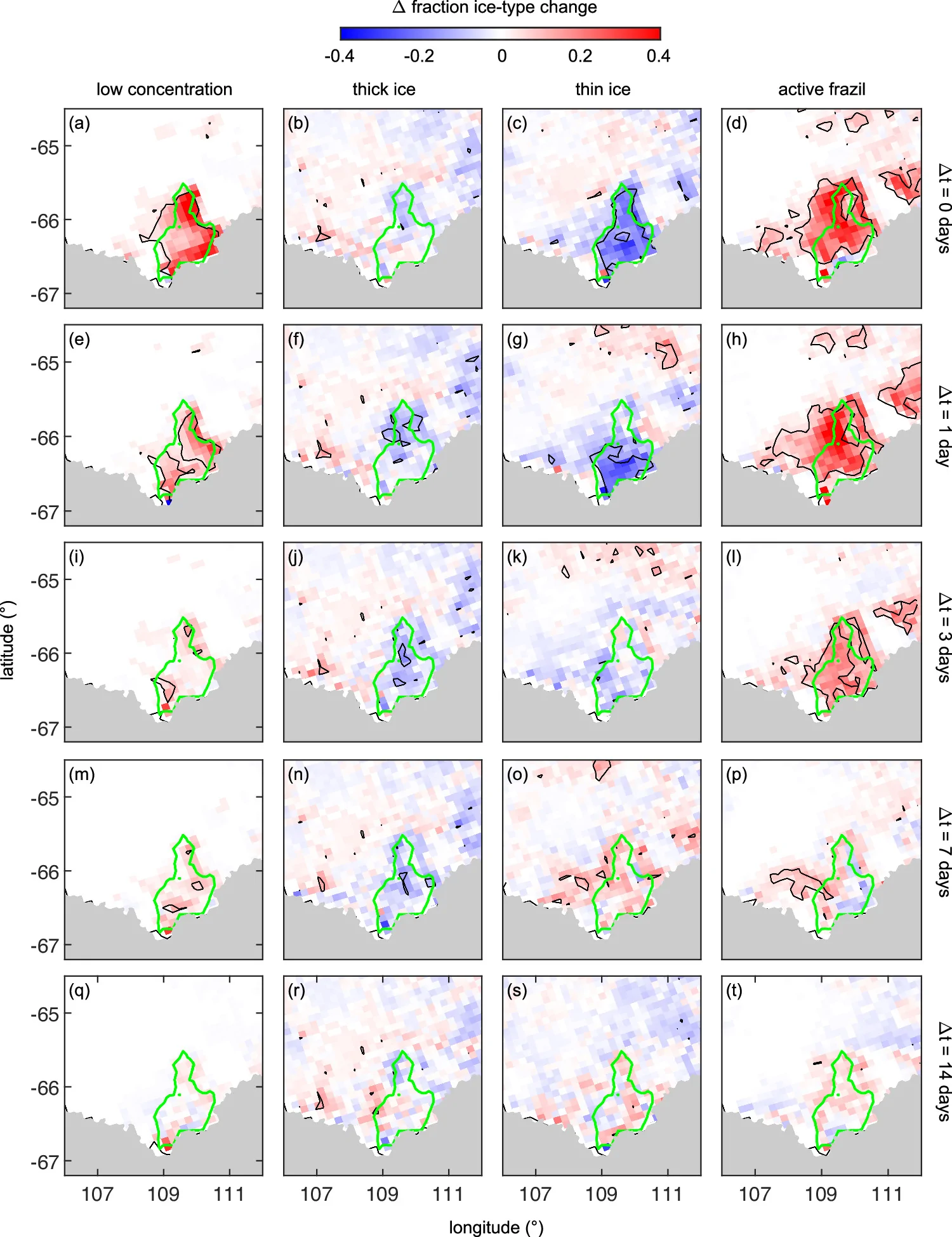
Ice-type changes following extreme events. Panels show the difference (*Δ* fraction of ice-type change) between conditionally-sampled extreme events of *I*_*br*_ and a randomly sampled subset. Colors indicate fractional transitions to a given ice-type category (for columns from left to right: low concentration (**a**, **e**, **i**, **m**, **q**), thick ice (**b**, **f**, **j**, **n**, **r**), thin ice (**c**, **g**, **k**, **o**, **s**), active frazil (**d**, **h**, **l**, **p**, **t**) with red indicating a relative increase in transition probability and blue indicating a relative decrease compared with the random subset. Rows correspond to different lags *Δt* relative to the extreme event. Black contours identify *p* < 0.05 relative to the random subset. The green contour identifies the region at which the polynya was present for at least 10% of the subset. A pronounced transition to active frazil is evident at *Δt* = 0 − 3 days, while thin ice shows a relative decline immediately after extremes and a tentative recovery after about a week.

The associated changes with extremes in *I*_*br*_ are also evident in ice-type transitions surrounding the polynya. Using the classification scheme of^29^, we calculate anomalies in the probability of transition to each ice type during extreme events relative to a randomly sampled subset. Extremes in *I*_br_ are characterized by a marked shift of the polynya surface toward active frazil ice (Fig. 9), indicating enhanced ice production and an expansion of the area actively forming new ice. A tentative reduction in the thin-ice fraction is observed immediately following these extreme events, with partial recovery after roughly one week. These patterns are qualitatively similar to those associated with extreme wind events, albeit with systematically lower probability anomalies (Suppl. Fig. S12).

## Discussion

In this study, we use rare field observations to demonstrate that energetic waves generated by strong winds from the Antarctic continent within the Vincennes Bay Polynya are steep enough to break up the surrounding sea ice. Analysis of a sea-ice concentration dataset spanning 2003–2022 reveals that such extreme events can substantially modify the surrounding ice cover. In particular, this inside-outward breakup, in contrast to the more commonly studied outside-inward break-up of pack ice by Southern Ocean swells, leads to a persistent reduction in concentration around the polynya that typically lasts at least one week. These prolonged anomalies are accompanied by shifts in ice type toward active frazil, implying enhanced new-ice formation and altered air-sea heat exchange that may influence both the extent and lifetime of the polynya.

The observed reduction in sea-ice concentration around the Vincennes Bay Polynya is most consistently explained by redistribution of the ice cover. During late winter, the surrounding fast ice and coastline restrict large-scale ice transport, including by the northward-extending fast-ice tongue and the fast ice to the west (Fig. 1), making export through the remaining downwind opening towards the northwest the likely driver of this redistribution. The reduction in ice thickness (Suppl. Fig. S11) and sea-ice concentration in and around the polynya is accompanied by statistically significant positive anomalies farther downwind of the polynya margins (Fig. 8). This pattern suggests a physically consistent redistribution of ice, possibly by localized rafting and compaction beyond the polynya edge^30–32^, consistent with downwind accumulation of exported ice. A similar pattern emerges in the sea-ice type product, where an increase in the occurrence of thick ice surrounds the polynya during periods of extreme values of the wave-induced break-up parameter compared to extreme wind events (Suppl. Fig. S11 and S12), further supporting the interpretation of wave-driven localized deformation and thickening. This interpretation is also supported by the in situ wave measurements from buoy B22, where attenuation inferred from peak-frequency downshifting suggests progressive consolidation of the sea ice during energetic wave events, with a reduced extent of low-concentration sea ice (Fig. 6), consistent with the satellite-derived pattern of localized thickening around the polynya.

The inferred thickening and positive concentration anomalies cannot be explained by wave-induced break-up alone, but indicate redistribution and compaction of the ice cover under convergent forcing. In this context, wave radiation stress provides a physically linked momentum source: as locally generated waves attenuate, their momentum is transferred to the ice through convergence of the wave radiation stress, which can drive ice drift, compaction, rafting and ice-edge deformation^30,31,33,34^. Our scaling estimates (see Methods) suggest that this wave-induced stress can be comparable to, or locally exceed, the wind stress close to the polynya ice edge, particularly under high-attenuation conditions (Suppl. Fig. S13). The estimated stress is also larger than typical estimates for longer-period swell in the outer MIZ^31^, reflecting the stronger momentum convergence associated with the rapid attenuation of short, locally generated waves. However, this same rapid attenuation confines the direct forcing to a relatively narrow band of the order of a kilometer around the polynya edge^34^. This relatively short forcing length scale does not preclude wave-induced fracture farther into the pack, as the rapid attenuation of shorter-wave components results in strong momentum transfer to the ice near the ice edge, whereas the remaining longer-period and more slowly attenuating waves may still be energetic enough to fracture the ice^30,35,36^. We therefore interpret the observed response as an interplay between wave-induced break-up, wave-induced momentum transfer and wind-driven redistribution: break-up preconditions the ice cover for redistribution by reducing its mechanical coherence and producing smaller, more mobile floes, while wind and wave-induced stresses provide the convergent forcing needed to transport, compact and raft the fragmented ice^30,37^.

Although the wave impacts observed in the Vincennes Bay Polynya are well supported, extending these conclusions to other coastal polynyas remains speculative without further in situ observations, as the dynamics between winds, waves, currents, and sea ice may vary considerably. For instance, grease ice can simultaneously attenuate waves and reduce dissipation from breaking, thereby sustaining wind-wave energy transfer^10^, and additional energy input may also occur within the pack itself^38^. In many coastal polynyas, strong winds render wave conditions duration rather than fetch-limited^39^. These complexities also help explain why wave models often struggle to replicate the observed wave fields: the relevant small-scale sea-ice properties, such as the distribution of grease, pancake, thin, and consolidated ice, are poorly constrained, yet they critically determine attenuation rates, breakup behavior, and the effective fetch. Without sufficiently resolved and well-characterized sea-ice conditions, particularly reliable ice-thickness observations, both the interpretation of wave observations and the fidelity of wave-ice model simulations remain fundamentally limited. Addressing these challenges will require more comprehensive in situ observations of both waves and the detailed sea-ice regime, underscoring the rarity and significance of the measurements presented here.

While winds are widely recognized as the primary driver maintaining open-water conditions in coastal polynyas, our results highlight that the waves generated by these winds play a critical and previously underappreciated role in this process. These findings emphasize the tightly coupled interactions between winds, waves, and polynyas, which remain insufficiently understood. As Antarctic polynyas have expanded in recent decades^40^, the influence of wave activity on sea ice by weakening the pack ice from within is likely to become increasingly important as effective fetches lengthen. Resolving these processes more accurately is essential for understanding not only the persistence of polynyas but also the evolving role of waves in shaping Antarctic sea ice.

## Methods

### In situ experiments

The wave measurements were obtained using open-source waves-in-ice buoys^16,17^. These instruments transmit GPS positions and one-dimensional wave energy spectra, *E*(*f*), derived from the measured vertical motion of the ice. We therefore restrict the analysis to non-directional bulk wave parameters, including significant wave height and peak period. Previous validation of these buoys indicates that they can resolve centimeter-scale wave motions and, under favorable conditions, millimeter-scale motions^16,17^. Following breakout, the buoys likely remained embedded in drifting ice fragments or floes, so the measurements are interpreted as the wave field experienced by the ice rather than as fully open-water wave conditions. As such, they may provide conservative estimates of the wave energy that would develop under equivalent open-water conditions. Since the buoys entered the polynya only following breakout from fast ice, these observations preferentially sample strong-forcing conditions and should not be interpreted as a random sample of all polynya wave states.

In total, four field experiments were conducted near Vincennes Bay (around 110.6^∘^ E, 66.2^∘^ S) between 2021 and 2025 to obtain in situ observations of waves, winds, and sea ice.

2021 deployment (B21): Four wave-ice buoys^16^ were deployed on 10 September 2021 on fast ice, three aligned parallel to the coast and one perpendicular. Two buoys were equipped with wind anemometers (6410 Anemometer, Davis Instruments) measuring mean wind speed 90 cm above the surface. Ice and snow thicknesses were 120–130 cm and 12–20 cm, respectively. One buoy drifted out following an early October breakout and transmitted until 17 November 2021.

2022 deployment (B22): Four OpenMetBuoys (OMBs)^17^ were installed on fast ice on 25 August 2022 along a transect approximately perpendicular to the ice edge (ice thickness 65–76 cm). Three ceased transmission within two weeks, likely due to snow accumulation. One buoy remained operational and drifted across the Vincennes Bay Polynya during a breakup event beginning 12 October, with maximum drift speeds around 1 m/s. Data transmission ended on 8 January 2023.

2024 deployment (B24): Three OMBs were deployed on 3 October 2024 along a transect perpendicular to the fast-ice edge. Ice and snow thicknesses were 55–92 cm and 14–30 cm, respectively. Two buoys drifted westward following breakup on 19 November, crossing the Vincennes Bay Polynya on 21 November with drift velocities up to 0.8 m/s. Transmission from one buoy ceased on 27 November; the second continued until 13 January 2025. As both exhibited similar trajectories and wave records, only the second buoy (B24) was analyzed further.

2025 deployment (B25): Four OMBs were deployed on 3 October 2025 along a transect perpendicular to the fast-ice edge. Ice and snow thicknesses were 60–62 cm and 11–17 cm, respectively. One buoy ceased transmitting after one week, while the remaining three drifted out of the fast ice on 1 November and crossed the Vincennes Bay Polynya on 2 November. Of these, we use only the buoy with the most complete and continuous record for analysis.

### Numerical simulation

To aid in the interpretation of the observations, we ran numerical simulations of ocean waves in the Vincennes Bay Polynya with the third-generation spectral wave model WAVEWATCH III (version 7.14,^22^,hereafter WWIII). The WWIII wave model solves the energy balance equation to predict the evolution and propagation of ocean surface waves:

1 $$\frac{{{{\rm{d}}}}E}{{{{\rm{d}}}}t}={S}_{{{{\rm{in}}}}}+{S}_{{{{\rm{ds}}}}}+{S}_{{{{\rm{nl}}}}}+{S}_{{{{\rm{bf}}}}}+{S}_{{{{\rm{ice}}}}},$$

where *E*(*f*, *θ*; ***x***, *t*) is the frequency-direction wave spectrum, and the RHS of Eq. (1) denotes different physical processes affecting wave energy, including wind input *S*_in_, dissipation owing to wave breaking and wave-induced turbulence production *S*_ds_, nonlinear four-wave resonant interaction *S*_nl_^41^, dissipation owing to bottom friction *S*_bf_^26^ and sea ice-related term *S*_ice_^23,42^. For our simulations, we specifically used the observation-based source term package ST6 for *S*_in_ + *S*_ds_^43^. An empirical frequency-dependent parameterization of ice-induced wave decay was adopted for *S*_ice_:

2 $${S}_{{{{\rm{ice}}}}}(f,\theta )=-{c}_{g}{c}_{i}\alpha (f)E(f,\theta ),$$

3 $$\alpha (f)={\beta }_{1}{f}^{2}+{\beta }_{2}{f}^{4},$$

where *c*_*g*_ is the group velocity, *c*_*i*_ is ice concentration, *β*_1_ = 2.12 × 10^−3^ and *β*_2_ = 4.59 × 10^−2^, respectively^44,45^. The parameterizations for other physical processes (i.e., *S*_nl_ and *S*_bf_) are the same as those used in ref. ^46^ and are not described here for the sake of brevity.

The spectral grid is discretized into 36 directions (*Δθ* = 10^∘^) and 35 exponentially spaced frequencies ranging from 0.037 to 0.953 Hz (*Δf*/*f* = 1.1). The WWIII regional model domain is bounded within 105^∘^–115^∘^E of longitude and 68^∘^–60^∘^S of latitude with a grid resolution of 0.05^∘^ × 0.05^∘^. The wind forcing we used was sourced from the ECMWF operational weather forecast (concatenating forecast hours 0–11 from each 12-h initialized forecast to create a complete set of forcings) with a spatiotemporal resolution of 1 h and  ~ 9 km. The ice concentration was based on satellite observations from the Advanced Microwave Scanning Radiometer 2 (AMSR2; 12-h and 3.125 km). The directional wave spectra at open-boundary points were provided by global runs at 0.25^∘^ × 0.25^∘^ using the same physics parameterizations^46^ and forced by ERA5 reanalysis winds^47^. The simulation periods for the cases of B21, B22, B24 and B25 are 1 September–1 November 2021, 1–31 October 2022, 1–30 November 2024, and 1 October–7 November 2025, respectively.

### Remote sensing

Several satellite products containing wave parameters were consulted, but at the time of break-out of the buoys remote sensing observations were limited. In this study, we were restricted to two Sentinel-1 synthetic aperture radar (SAR) images taken around the time of break-out of B21 and B24, with the observation of B21 being in the middle of the ice pack, a few hundred kilometers away from the buoy, and the satellite observation of B24 passing over the exact location of the buoy (Suppl. Fig. S10). We limit ourselves to the manual retrieval of the peak wavelength *λ*_*p*_, visible as bright bands in the images due to velocity bunching^31^. These estimates of *λ*_*p*_ are meant as an indicator of the expected low-frequency peak of the wave field.

We further used MODIS satellite imagery (https://worldview.earthdata.nasa.gov/, last access: 26 May 2025) together with AMSR-E and AMSR2 sea-ice concentration data derived using the ARTIST Sea Ice (ASI) algorithm^48^, downloaded from the University of Bremen sea-ice data archive (https://data.seaice.uni-bremen.de/; last access: 20 May 2026), to interpret sea-ice conditions during instrument deployment and to provide context for the wave observations. In addition, we used SMOS sea ice thickness^49^, downloaded from https://data.seaice.uni-bremen.de/smos/(last access: 22 February 2024), and AMSR2 Thin Ice Thickness (TIT) Level 3 data (ver. 100) provided by the Japan Aerospace Exploration Agency / Earth Observation Research Center (JAXA/EORC), https://www.eorc.jaxa.jp/AMSR/research/(last access: 25 September 2025). Ice types were classified following^29^ into four categories: low concentration, thick ice, thin ice, and active frazil. In this framework, active frazil represents mixed frazil, grease, pancake ice, and intermittent open water formed under strong winds, while thin ice refers to relatively uniform nilas or thin level ice formed under calmer conditions, allowing us to interpret the ice regime in and around the Vincennes Bay Polynya.

### Fetch-limited growth relations

In the absence of ice and swell, wave conditions in a polynya are expected to be constrained by wind speed and fetch. Under such conditions, we may estimate the significant wave height and peak wavelength based on the fetch-limited growth conditions where $\chi=gF/({U}_{10}^{2})$, $\varepsilon=(1/16){g}^{2}{H}_{s}^{2}/({U}_{10}^{4})$, and *υ* = *f*_*p*_*U*_10_/*g* are the normalized fetch, wave energy, and frequency, respectively. There, *g* is the gravitational acceleration, *U*_10_ is the wind speed at 10 m reference height, *f*_*p*_ the peak frequency, and *F* is the fetch (the distance to the upwind polynya edge). For fetch-limited wind seas, the normalized wave energy and peak wave period are related to the normalized fetch as^25,50^:

4 $$\varepsilon=5.18\times 1{0}^{-7}{\chi }^{0.90},$$

5 $$\varepsilon=7.2\times 1{0}^{-6}{\upsilon }^{-3.33}.$$

6 $$\upsilon=2.18{\chi }^{-0.27},$$

### Wave-induced sea ice break-up

We estimate the break-up potential of sea ice by waves based on the strain failure criterion^18,51^:

7 $${I}_{br}=\frac{\frac{1}{2}{H}_{s}hY}{\sigma {\lambda }_{p}^{2}},$$

where *H*_*s*_ is the significant wave height, *h* is the sea ice thickness, *Y* is the Young’s modulus, *σ* is the sea ice flexural strength, and *λ*_*p*_ is the peak wavelength. It was found that *I*_*br*_ = 0.014 is a good estimator of the wave-induced break-up threshold^18^.

While Eq. (7) is supported by field observations, the criterion becomes physically unrealistic for very short waves. For example, short waves with very small amplitudes would still be able to break very thick ice. Instead, short wave energy is unlikely to flex thick ice as most of their energy would be reflected or turned into elastic deformation. Consequently, the motion of the surface in the presence of sea ice is not the same as the motion of the surface in open water. Following conservation of energy in the wave-ice system,^52^ introduced the ratio *G* based on the work of^53^, and one may then define the break-up parameter as:

8 $${I}_{br}=\frac{\frac{1}{2}{H}_{s}hY}{G\sigma {\lambda }_{p}^{2}},$$

where $G=1+D{k}_{p}^{4}$, with *D* = *Yh*^3^/(12*ρg*(1 − ν^2^)), *ρ* is the water density, *k*_*p*_ is the peak wave number, and ν is Poisson’s ratio.

One may estimate the wave length *λ* = 2*π*/*k* from the flexural-gravity dispersion relation in deep water of^54^:

9 $${\omega }^{2}=\frac{gk(1+D{k}^{4}-Q{k}^{2})}{1+Mk}.$$

where *M* = *hρ*_*i*_/*ρ*, *ρ*_*i*_ is the density of sea ice, *Q* = *Ph*/(*ρg*) and *P* is the compressive stress. Given the scarcity of data on the mechanical properties of sea ice in the study area, we use *σ* = 0.4 × 10^6^ Pa, ν = 0.3 and *Y* = 3 × 10^9^ Pa, as indicative values^18,55–57^, which are commonly adopted for sea ice, and we further assume compressive stresses to be negligible, i.e., *P* = 0. The choice of ice thickness is discussed in the 'Conditional averaging' section.

To estimate *H*_*s*_ and *λ*_*p*_ we make use of the fetch-limited growth relations (4)–(6), which are a function of fetch *F* and wind speed *U*_10_. To estimate the fetch, we make use of the polynya edge dataset of^27^, which is based on the sea ice concentration criterion of 60% using AMSR-E and AMSR2 data. The dataset consists of daily estimates of Antarctic polynya edges from 2003–2022, for the months of April to October. As the fetch *F* is defined as the along-wind distance over which the wind transfers energy to the waves, we define the fetch here as the distance between the upwind and downwind edge of the polynya (i.e., the dimension of the polynya along the direction of the wind), with representative wind direction and wind speed calculated from ERA5 reanalysis data^47^ over the 24 h period from midday on the preceding day to midday on the analysis day.

We note that the winds provided by ERA5 generally underestimate the extreme winds at the site^46,58^. For example, in Suppl. Fig. S14, we compare the measured winds by two buoys deployed on fast ice in 2021 (corrected to 10 m height) against ERA5 and the wind speed measured on land at Casey Station. We observe overall good agreement between the winds measured by the anemometer attached to the buoys and the anemometer based on land (Suppl. Fig. S14c). To ensure reasonable interpretation of the buoy data when comparing against the fetch-limited wave growth relations (i.e., Eqs. (4) and (6)) we show estimates derived from both the anemometer measurements on land, as well as ERA5 winds. However, for the long-term estimates of *I*_*br*_ in this study, we make use of ERA5 winds.

### Wave-induced stress on sea ice

We estimate the stress exerted on the ice by attenuating waves following^31^, assuming normally incident waves:

10 $${\tau }_{w}(x)=-\frac{1}{2}{\rho }g\int\frac{dE(f,x)}{dx}\,df.$$

where *E*(*f*, *x*) is the wave variance density spectrum _*w*_. With

11 $$E(f,x)=E(f,0)\exp [-\alpha (f)x],$$

this gives the wave-induced stress magnitude

12 $${\tau }_{w}(x)=\frac{1}{2}{\rho }g\int\alpha (f)E(f,x)\,df.$$

Here, *E*(*f*, 0) is represented by a JONSWAP spectrum with peak enhancement factor *γ* = 3.3, using *f*_*p*_ and *H*_*s*_ derived from the fetch-limited wave growth relations.

For comparison, the wind stress magnitude is approximated as

13 $${\tau }_{a}={\rho }_{a}{C}_{D}{U}_{10}^{2},$$

where *ρ*_*a*_ is the air density and *C*_*D*_ is the air–ice drag coefficient, taken here as 1.45 × 10^−3^ ^31^.

### Conditional averaging

To evaluate the broader sea ice response to wave-induced breakup, we computed conditional means of sea ice concentration (*c*_*i*_) associated with extreme breakup potential (*I*_*br*_). Extreme wave conditions were defined as *I*_*br*_ > *I*_*br*,0.95_, where *I*_*br*,0.95_ is the 95th percentile of the August–October dataset (Fig. 7). Equivalently, one can define extreme wind conditions as *U*_10_ > *U*_10,0.95_, for which *U*_10,0.95_ = 15.2 m s^−1^. However, as extreme *I*_*br*_ events necessarily require strong winds as well as sufficient fetch, a comparison with wind extremes alone does not fully separate the effect of winds from locally generated waves. We therefore instead considered high-wind conditions defined by *U*_10_ > *U*_10,0.90_, where *U*_10,0.90_ = 13.6 m s^−1^, and separated these into short- and long-fetch subsets using the median fetch of the high-wind subset (*F* = 34 km). This allows the sea ice response to be compared between events with broadly similar wind forcing, but different potential for fetch-limited wave growth (Suppl. Fig. S15). We note that the wind conditions in each of these subsets are broadly similar in magnitude and direction (Suppl. Fig. S16). The analysis was restricted to August–October, when the polynya is fully developed and its extent is less affected by background ice growth.

The relative change in sea ice concentration was quantified as the anomaly *Δc*_*i*_ = *c*_*i*_(*t*_0_ + *Δt*) − *c*_*i*_(*t*_0_ ﻿− *t*_ref_), where *t*_0_ is the time of the extreme event and *t*_ref_ represents the baseline condition preceding the event. A randomly selected subset was used as a control to distinguish wave-driven anomalies from seasonal variability. Statistical significance was assessed via a two-sided permutation test (2000 iterations).

To determine an appropriate value for *t*_ref_, we consider the timescale where the relevant atmospheric and oceanic forcings become decorrelated. We analysed the temporal autocorrelation of ERA5 10-m wind speeds between 2003–2022 across the Vincennes Bay Polynya between 65–66 ^∘^S and 108–109 ^∘^E. For each grid cell at 0.25^∘^ interval, we computed the autocorrelation function *R*(*τ*) of the wind speed time series, with *τ* being the time lag. The effective decorrelation time was defined as the first zero crossing *τ* ≈ 3.3 days (Suppl. Fig. S17). This metric represents the point beyond which the winds can no longer be considered statistically correlated with the extreme events. As waves are generated by the wind, we use this as the general value of *t*_ref_ rounded to the closest integer, i.e., *t*_ref_ = 3 days. We note that *t*_ref_ = 4 days does not affect the interpretation of the results, as this timescale exceeds the autocorrelation time.

Sea ice at the polynya edge is typically thin (*h* ~ 0.16 m^28^) and thickens progressively with distance into the pack. Although *h* ~ 0.16 m represents the local edge thickness, such a value produces the largest *I*_*br*_ for short-period waves (Eq. (8)). These short waves are indeed efficient at breaking thin ice immediately at the edge but attenuate rapidly and therefore have limited influence deeper into the pack, where the ice is thicker. For example, given a typical attenuation rate for a 3–4 s period wave, 95% of the energy would be attenuated within a distance of 0.5–10 km, for high and low attenuation rates respectively. Using the local edge thickness therefore favors short-wave events with limited penetration, which is inconsistent with our aim of identifying wave conditions with broader spatial impact.

To capture wave events with greater spatial impact, we therefore consider a higher, more representative ice thickness for such an area. Here we consider *h* ≈ 0.5 m, for which the extremes correspond to longer-period waves of about 6 s. These waves attenuate less rapidly than shorter-period waves, penetrate farther into the pack, and still exceed the break-up threshold for thin ice near the polynya edge. This thickness corresponds to the transition from newly formed to consolidated ice, consistent with SMOS 50th-percentile thicknesses ( ~ 0.5 m) typically observed 50–60 km from the ice edge at the Vincennes Bay Polynya. Taking *α*_*H*_ = 1.3 × 10^−1^*f*^2.66^ ^19^ and *α*_*L*_ = 2.12 × 10^−3^*f*^2^ + 4.59 × 10^−2^*f*^4^ ^44^ as representative high- and low-attenuation bounds, respectively, we estimate that such waves may remain dynamically relevant over distances of approximately 10–80 km into the pack.

We verified that our results are not sensitive to the exact choice of *h* by quantifying the subset stability of the extreme events using the Jaccard index *J*. Across *h*_ref_ ∈ [0.3, 0.7] m, *J* plateaus at *J* ≳ 0.8 for *h* ≳ 0.5 m, indicating a high degree of overlap ( ~ 80%) among the identified extreme-event subsets across this range (Suppl. Fig. S18).

To examine how extreme wave and wind events influence the surface ice composition, we extended the conditional-averaging framework to satellite-derived ice-type classifications from^29^, distinguishing between low-concentration, thick-ice, thin-ice, and active-frazil categories. For each event subset (extreme *I*_br_, extreme *U*_10_, and randomly selected controls), we identified all grid cells where the ice type changed from any other category to a given target class (low-concentration, thick ice, thin ice, or active frazil) between *t*_0_ ﻿− *t*_ref_ and *t*_0_ + *Δt*. Each transition was assigned a value of one, while non-transitions were assigned zero, such that the temporal mean represents the probability of transition to that ice type. To isolate the event-driven response from background seasonal variability, we computed the anomaly by subtracting the corresponding random subset from the *I*_br_ and *U*_10_ results. The resulting fields therefore describe the spatial anomaly in the probability of transition to each ice type, with positive (negative) values indicating an increased (decreased) likelihood of transition relative to background conditions. Statistical significance was assessed using the same permutation approach as for sea ice concentration.

## Supplementary information

Supplementary Information

Transparent Peer Review file

## Data Availability

All observational buoy data generated in this study are archived at the Australian Antarctic Data Centre and are publicly available under the following accession codes: (https://doi.org/10.26179/ctcy-f258) (2021, 2022 and 2024 buoy observations); (https://doi.org/10.26179/gsb8-af74) (2025 buoy observations). ERA5 reanalysis data are available from the Copernicus Climate Data Store (ECMWF, C3S), accessed on 30 April 2025. AMSR-E and AMSR2 sea-ice concentration and SMOS sea-ice thickness data used in this study are available from the University of Bremen sea-ice data archive (https://data.seaice.uni-bremen.de/). AMSR2 TIT L3 (ver. 100) data were provided by the Japan Aerospace Exploration Agency / Earth Observation Research Center (JAXA/EORC), accessed on 25 September 2025. MODIS imagery was accessed via NASA’s Worldview application (https://worldview.earthdata.nasa.gov), part of the NASA Earth Science Data and Information System (ESDIS), on 26 May 2025. Sentinel-1 data are available from the European Space Agency (ESA) via the Copernicus Open Access Hub, accessed on 26 May 2025. Meteorological wind data at Casey station were provided by the Bureau of Meteorology, ⓒCommonwealth of Australia, and are licensed under the Creative Commons Attribution 4.0 International licence. Additional visualization and access to satellite products were supported by the Ocean Virtual Laboratory (OceanDataLab, https://ovl.oceandatalab.com).

## Code availability

The code provided with this manuscript consists of MATLAB analysis scripts used to process the observational, model, and satellite datasets and reproduce the quantitative results and figures. It is publicly available at (https://doi.org/10.5281/zenodo.20316406), together with a description of the dependencies, input data, and reproduction steps.
