# Supplementary material for: Inside-outward breakup of sea ice by locally generated waves in the Vincennes Bay Polynya
Source: Nat Commun. 2026 Sep 29;17:11224. doi: 10.1038/s41467-026-77979-y (PMC13720198; doi:10.1038/s41467-026-77979-y)
Supplement: Supplementary file 1 [file 41467_2026_77979_MOESM1_ESM.pdf]

Supplementary Information for:
Inside-outward breakup of sea ice by locally generated waves in the Vincennes Bay Polynya

Joey J. Voermans, Qingxiang Liu, Lang Cao, Josh Kousal, Petra Heil, Alexander D. Fraser, Clarence O. Collins, Jean Rabault, Alexander V. Babanin

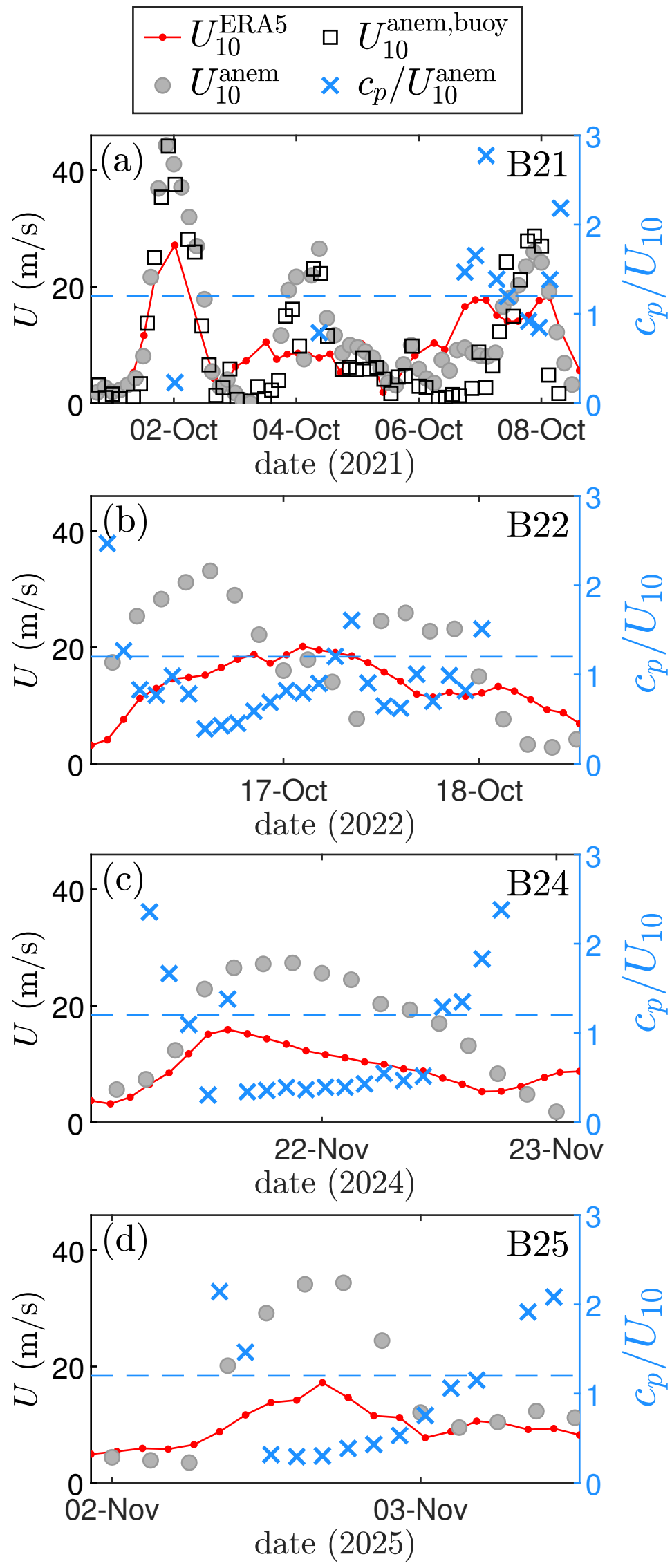

Fig. S1: Comparison between ERA5 wind speed (U_{10}^{ERA5}) and in-situ anemometer measurements at Casey Station (U_{10}^{anem}), and anemometer measurements by two buoys deployed on fast ice ($U_{10}^{\mathrm{anem,buoy}}$), around the time of the polynya crossing. The ratio of wave phase speed and wind speed is provided in blue, where $c_p/U_{10} < 1.2$ typically indicates wind waves, and $c_p/U_{10} > 1.2$ swell.

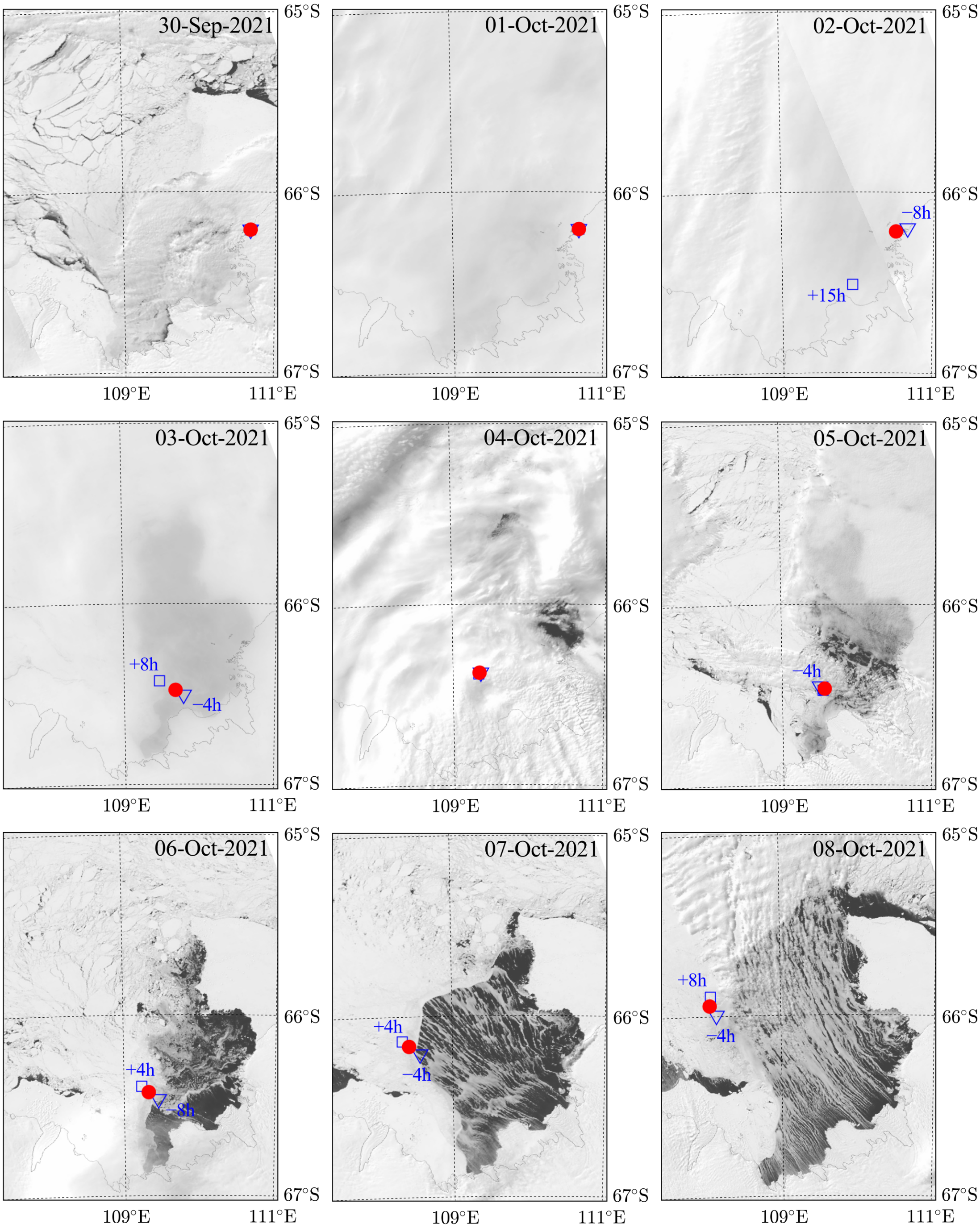

Fig. S2: MODIS satellite images around the time of the polynya crossing by B21. Red solid circle represents the buoy location at around the time of the image, with the blue triangle and square the received locations just before and after the red solid circle, respectively. The time differences between observations are given when drift was noticeable at that time.

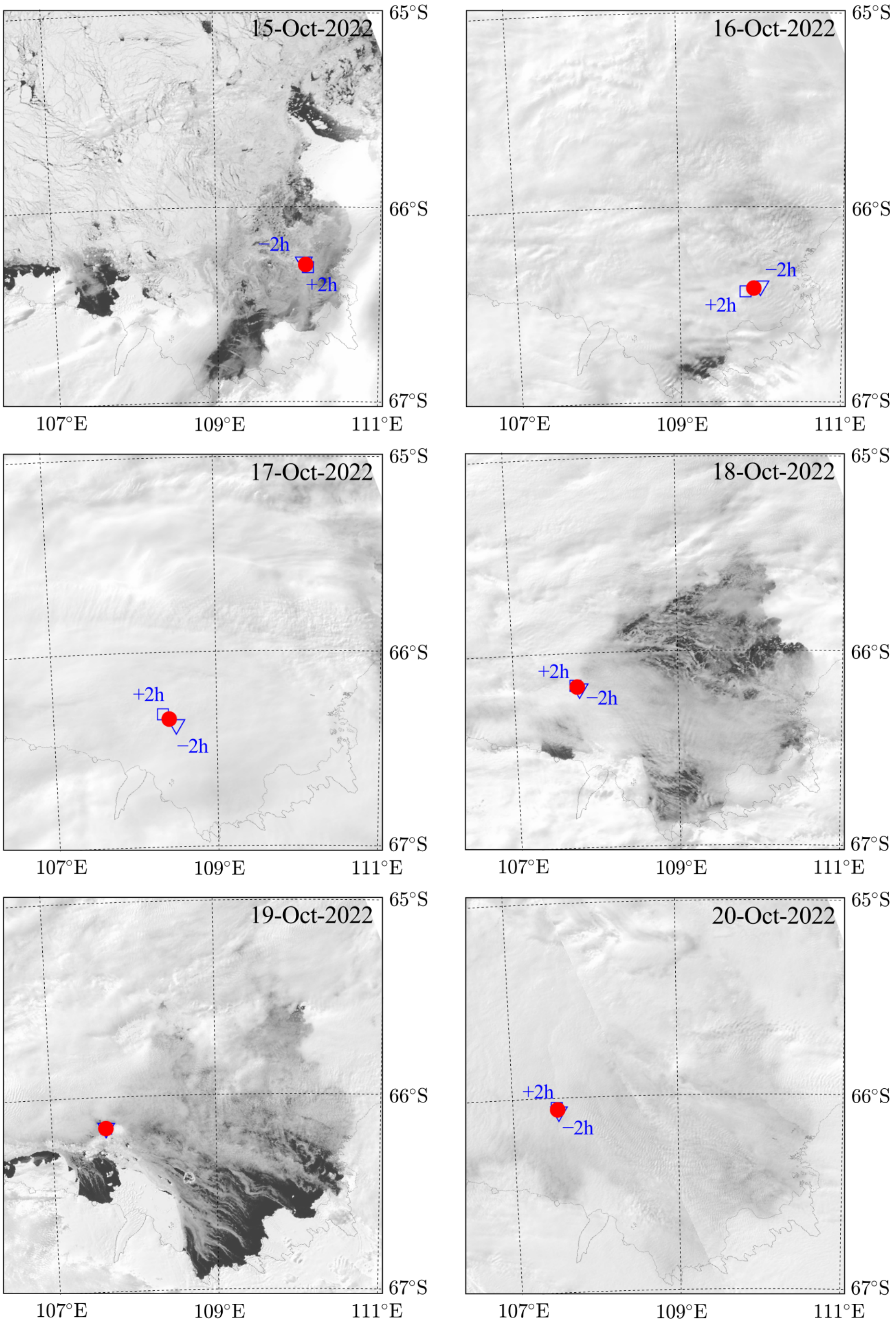

Fig. S3: Same as Fig. S2, but for B22.

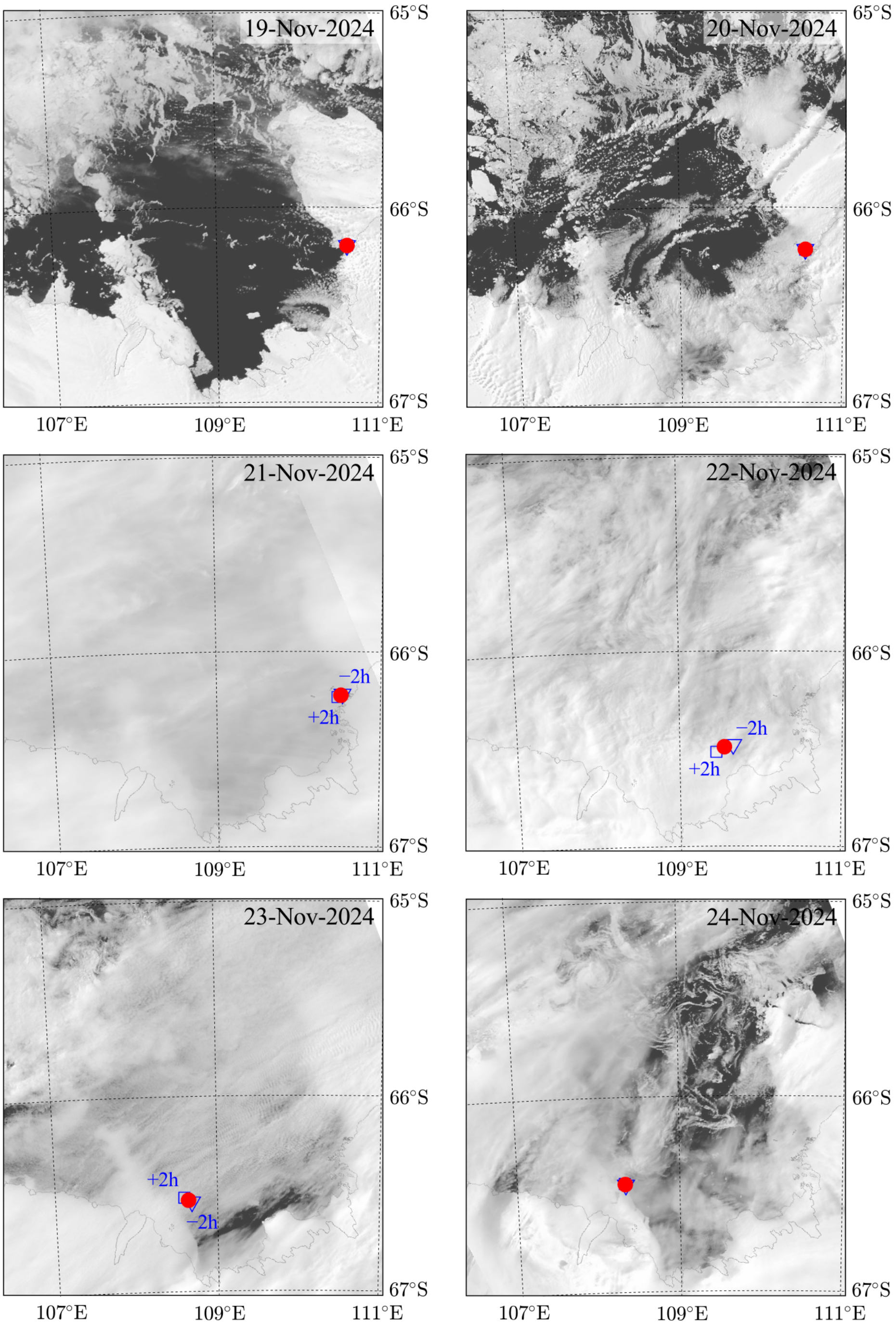

Fig. S4: Same as Fig. S2, but for B24.

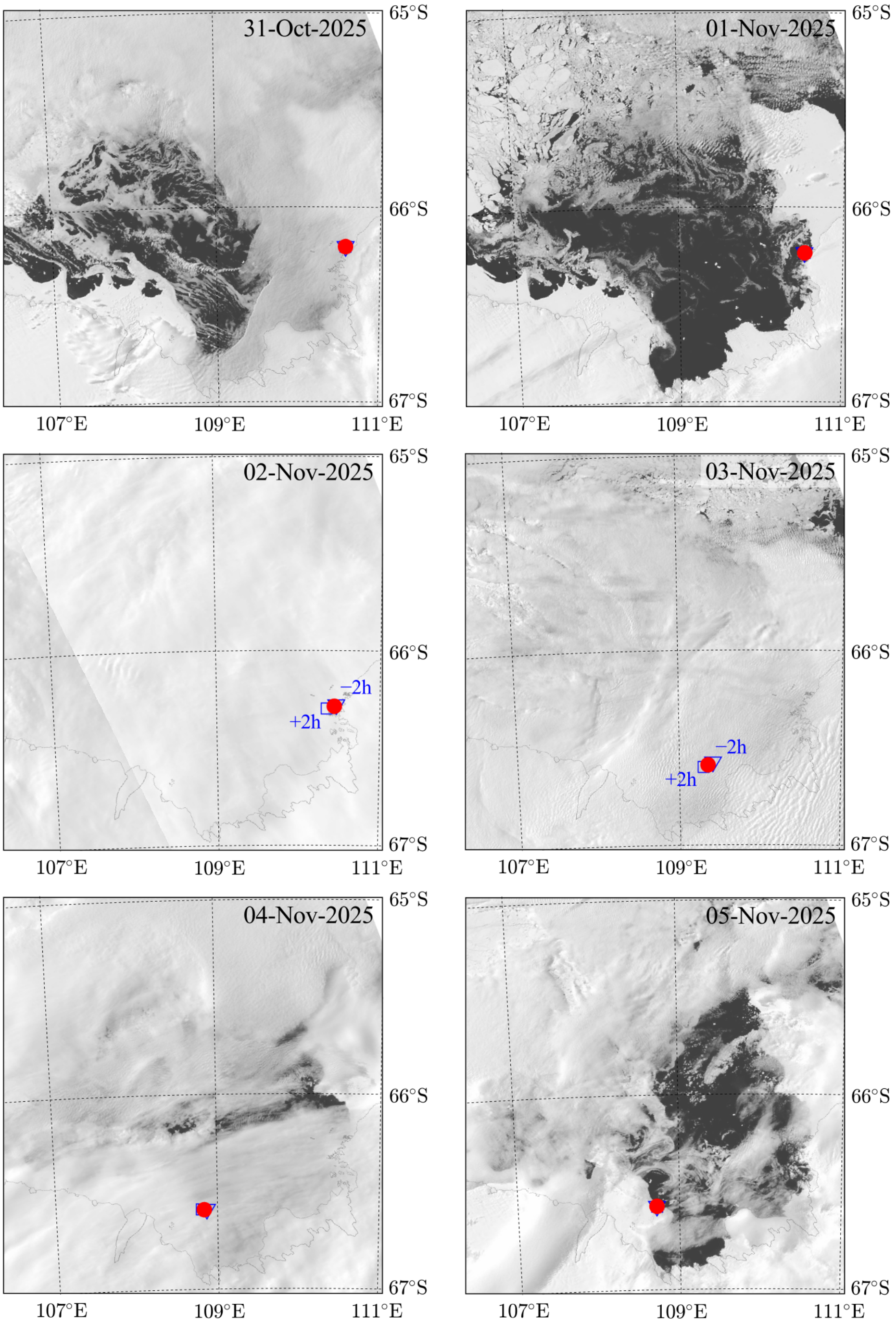

Fig. S5: Same as Fig. S2, but for B25.

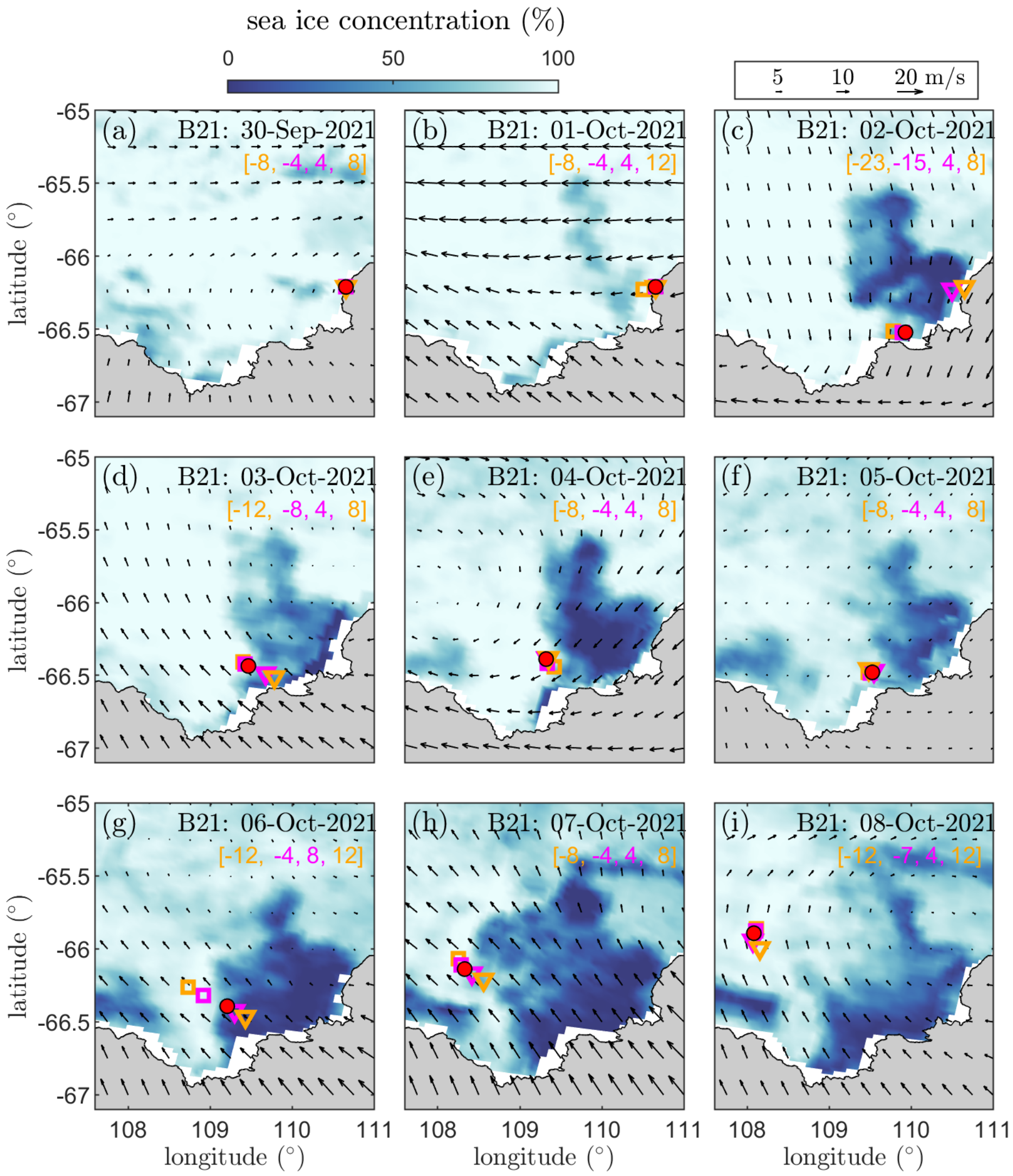

Fig. S6: AMSR2 sea-ice concentration around the time of the polynya crossing by buoy B21. Arrows indicate ERA5 winds. The red circle marks the buoy location at 12:00 on each day, while the orange triangle, magenta triangle, magenta square and orange squares denote the two transmission points immediately before and after, respectively. The relative timing of these transmissions is shown in the top right corner of each subplot.

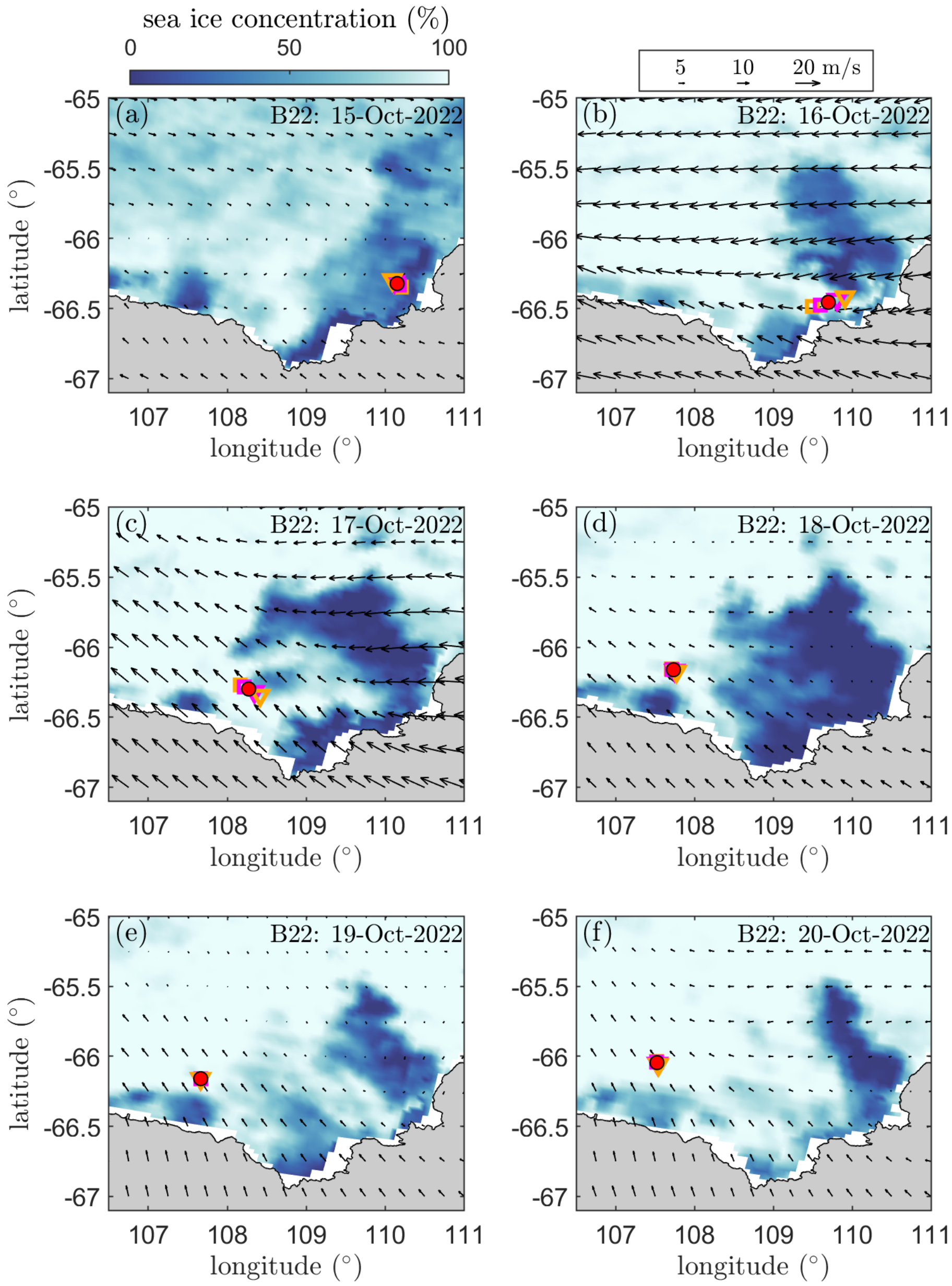

Fig. S7: Same as Fig. S6 but for B22. Triangles and squares are positions -4, -2, 2 and 4 hours relative to the buoy's location at the red solid circle.

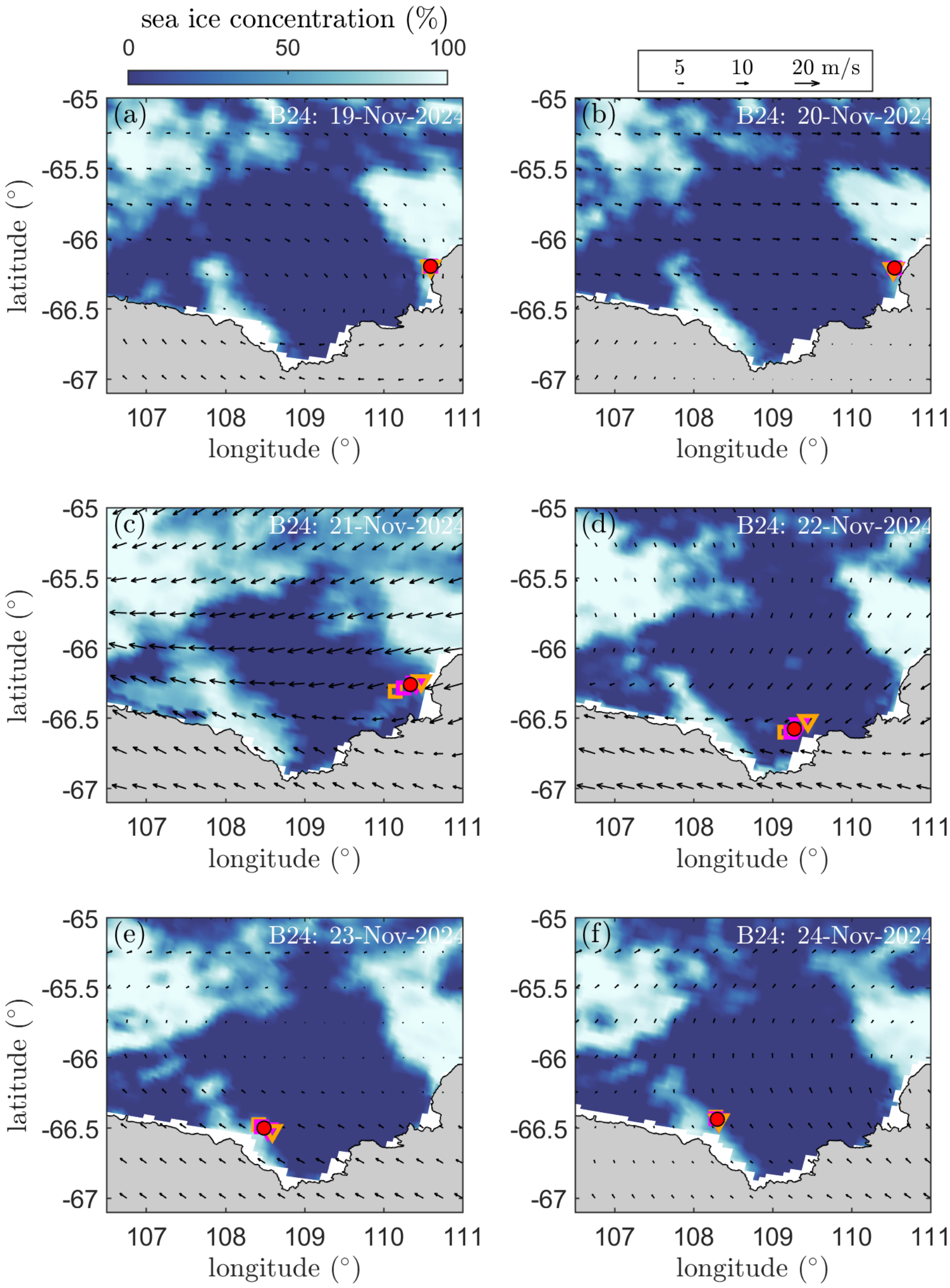

Fig. S8: Same as Fig. S6 but for B24. Triangles and squares are positions -4, -2, 2 and 4 hours relative to the buoy's location at the red solid circle.

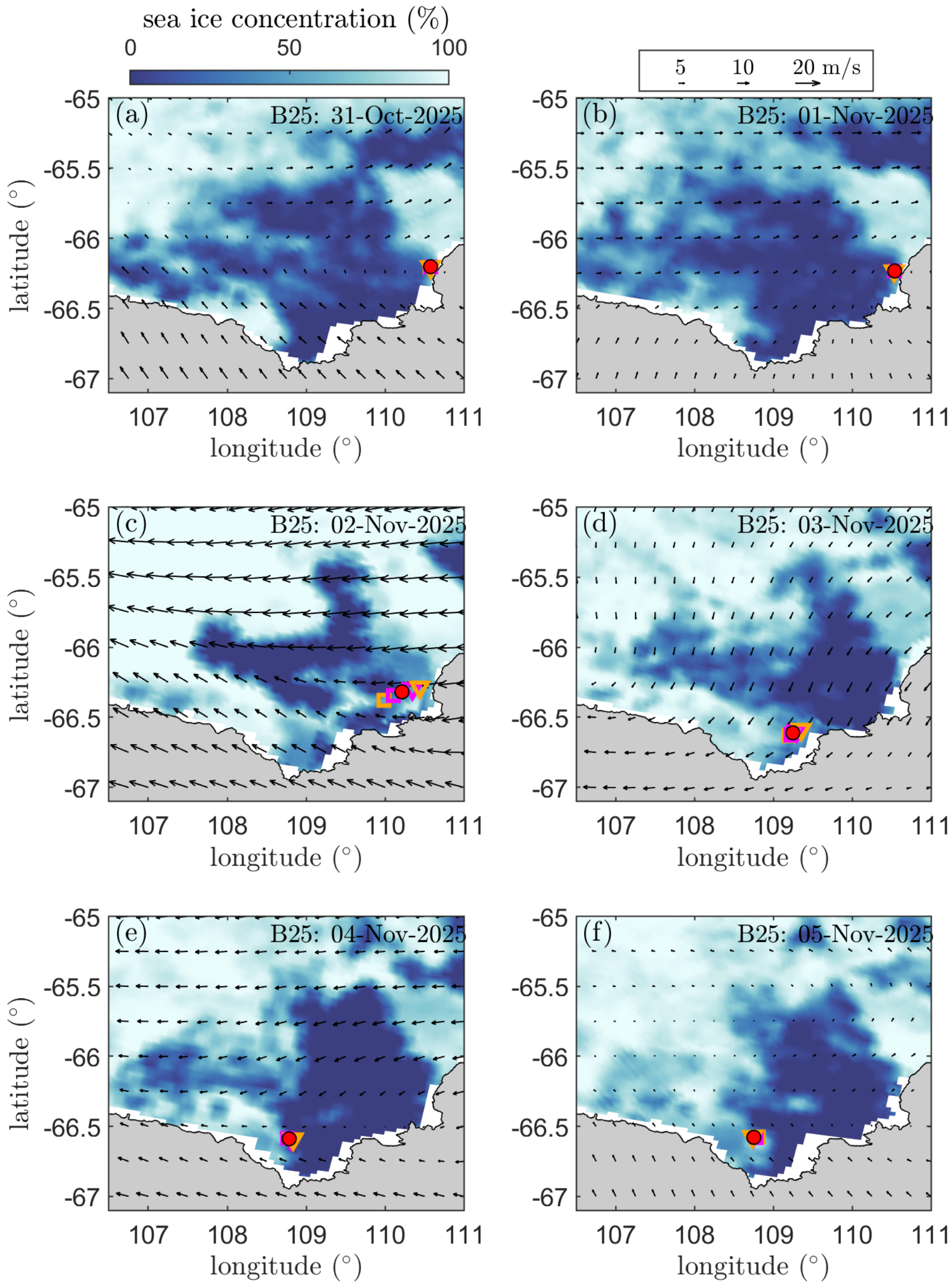

Fig. S9: Same as Fig. S6 but for B25. Triangles and squares are positions -4, -2, 2 and 4 hours relative to the buoy's location at the red solid circle.

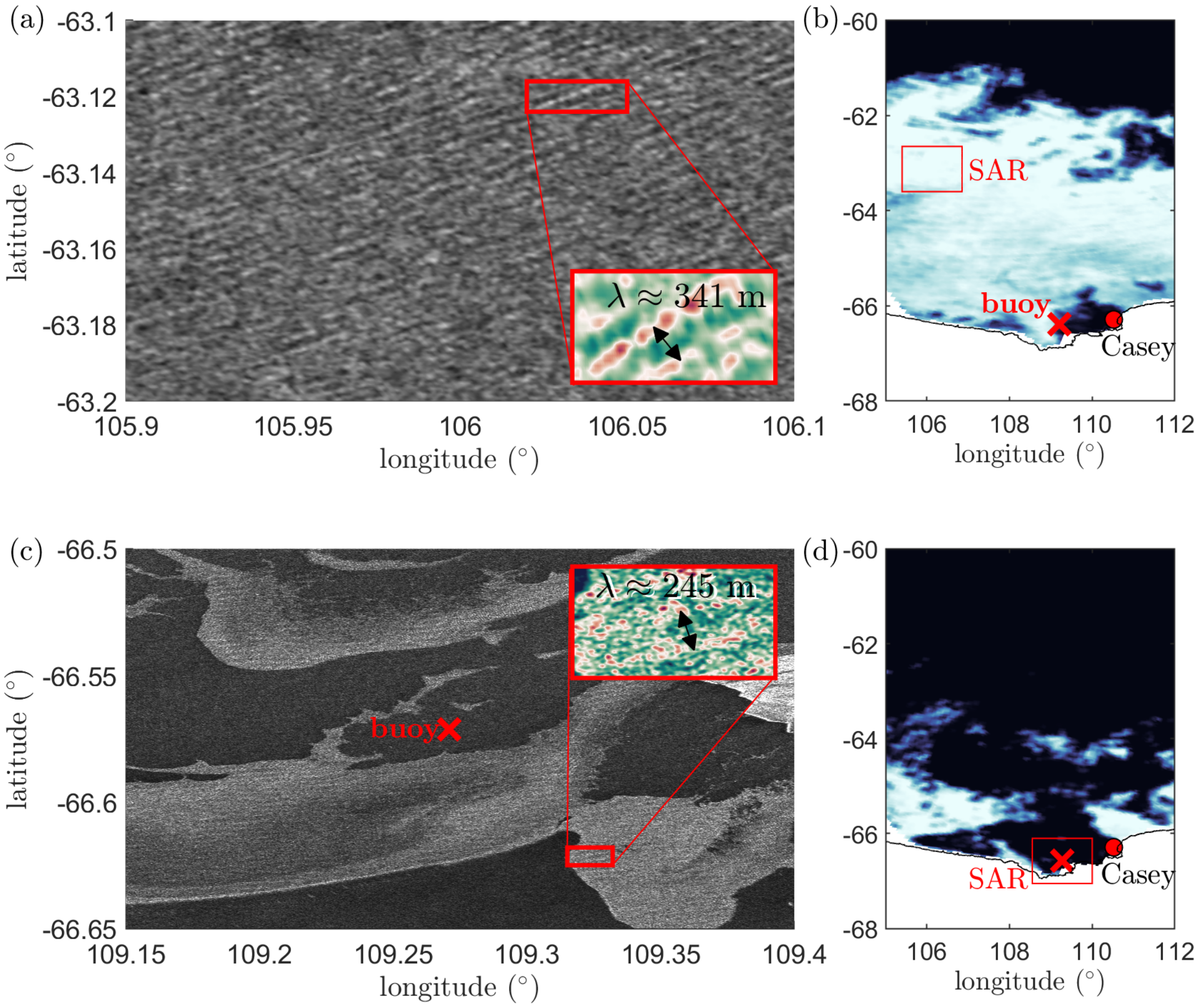

Fig. S10: Sentinel-1 synthetic aperture radar (SAR) imagery showing visible surface gravity waves around the time of polynya crossings of (a) buoy B21 on 6 October 2021 and (c) buoy B24 on 22 November 2024. Buoy locations are marked by the red crosses. Estimated peak wave length is $\lambda_p \approx 341$ m for B21, and $\lambda_p \approx 245$ m for B24. Corresponding large-scale AMSR2 sea ice concentration fields are shown in (b) and (d), respectively. SAR data correspond to file IDs S1A_EW_GRDM_1SDH_20211006T122932_20211006T123032_039998_04BC0B_5746_EC and S1A_IW_GRDH_1SSH_20241122T125309_20241122T125337_056667_06F3D1_564C_EC, respectively, and retrieved via the Copernicus Open Access Hub.

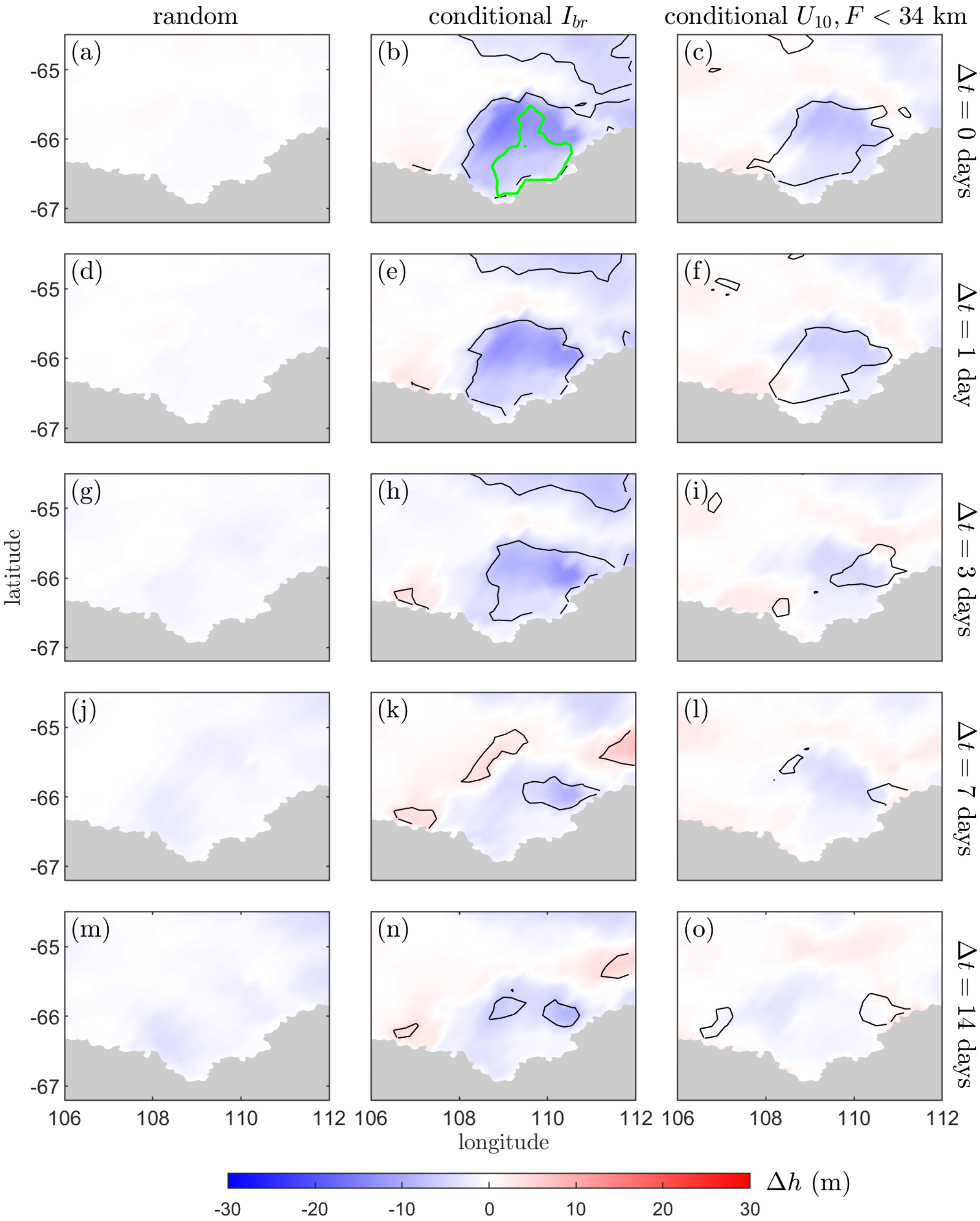

Fig. S11: Conditionally-averaged sea ice thickness (SMOS) difference maps for August–October, relative to three days before the samples. The first column is based on random sampling, while the second and third columns are sampled based on extremes in I_{br} and high-wind, short-fetch conditions, respectively. The short-fetch subset was derived by selecting events exceeding the 90th percentile of U_{10} and with fetch below the median fetch length ($F = 34$ km). Rows represent the number of days after the extreme event Δt. Black contours in the second column identify $p < 0.05$ relative to the baseline, and in the third column relative to the conditionally-sampled mean of I_{br}. We note that the thin-ice retrieval is limited to a maximum thickness of 0.5 m, which may introduce a bias in the difference maps.

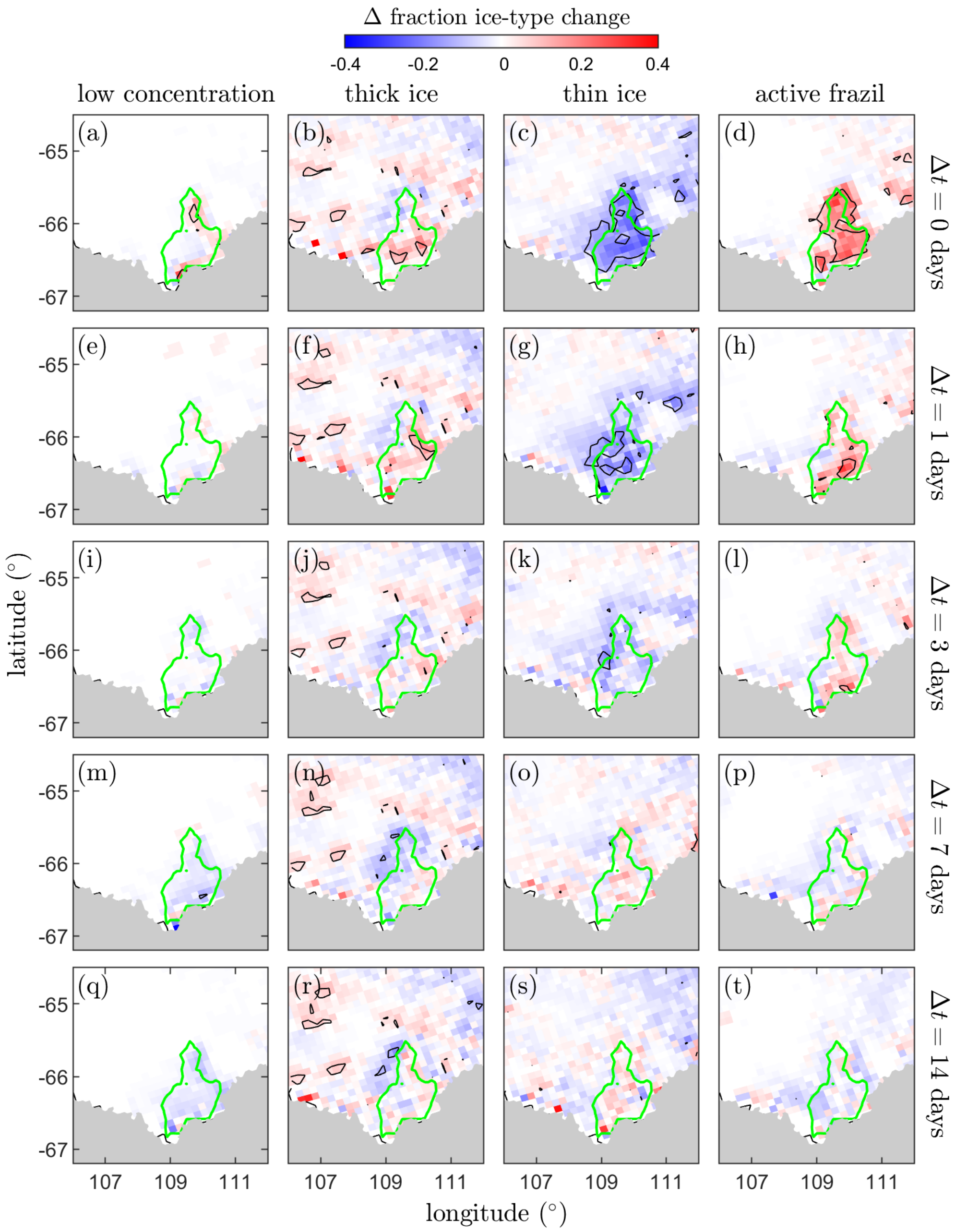

Fig. S12: Anomalies in ice-type transition probability during high-wind, short-fetch events relative to baseline conditions. Panels show the difference in the fraction of transitions to each ice-type category between the extreme-U_{10} subset and a randomly sampled subset. The short-fetch subset was derived by selecting events exceeding the 90th percentile of U_{10} and with fetch below the median fetch length ($F = 34$ km). Columns correspond, from left to right, to transitions to low concentration, thick ice, thin ice, and active frazil. Red indicates an increased transition probability during extreme-wind events relative to the random subset, while blue indicates a decreased probability. Rows represent the lag Δt relative to the extreme event. Black contours indicate $p < 0.05$ relative to the random subset. The green contour identifies the region in which the polynya was present for at least 10% of the extreme-I_{br} subset.

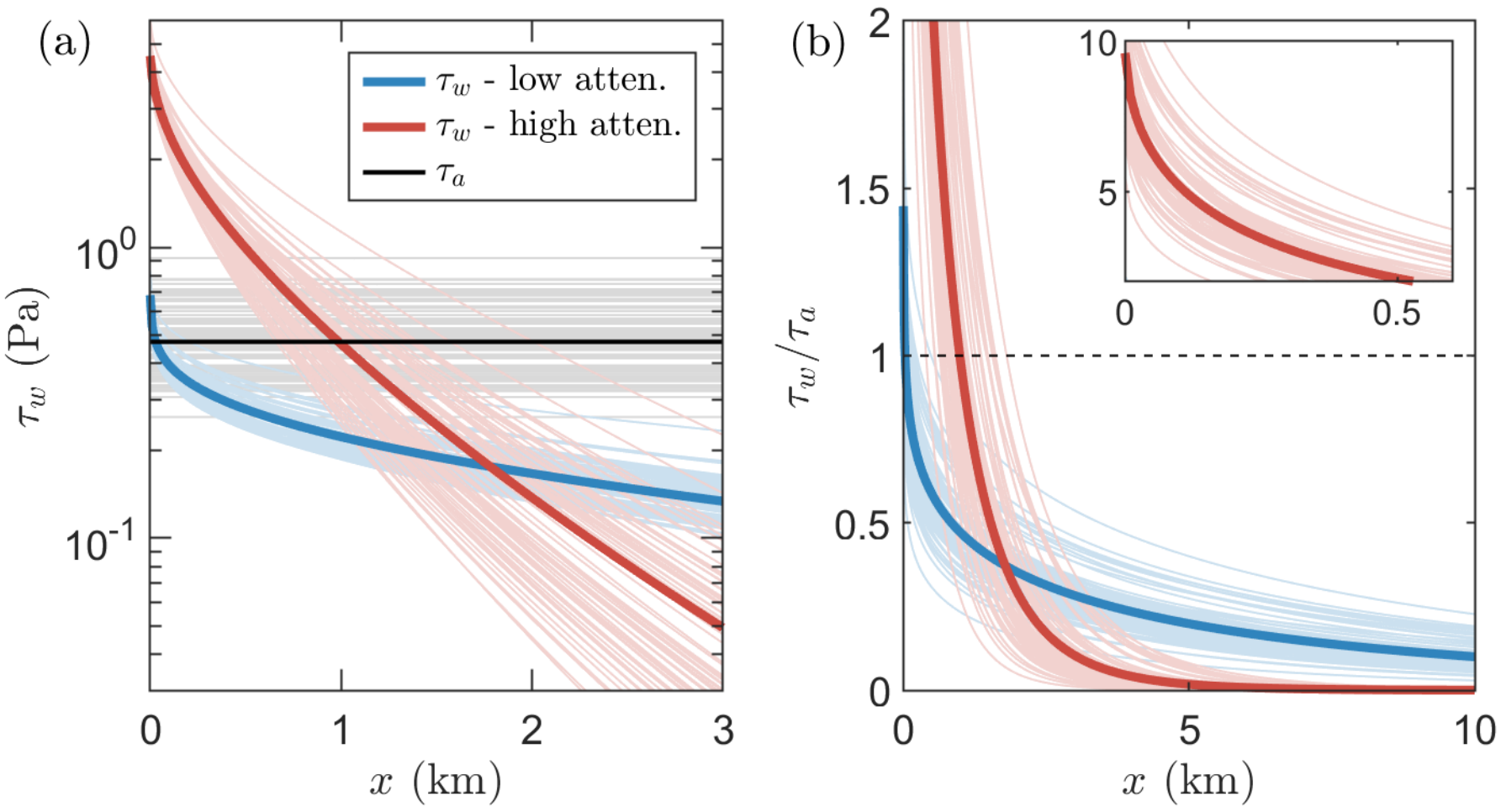

Fig. S13: Comparison of the estimated mean on-ice wave stress τ_w for low (blue) and high (red) wave attenuation rates against mean wind stress τ_a (black), with lighter shades showing the individual estimates for the polynya subset. Dimensional and non-dimensional estimates are provided in (a) and (b), respectively. Dashed line in (b) corresponds to $\tau_w/\tau_a = 1$ For low and high attenuation rates, we use $\alpha_L(f) = 2.12 \times 10^{-3} f^2 + 4.59 \times 10^{-2} f^4$ [1] and $\alpha_H = 1.3 \times 10^{-1} f^{2.66}$ [2], respectively.

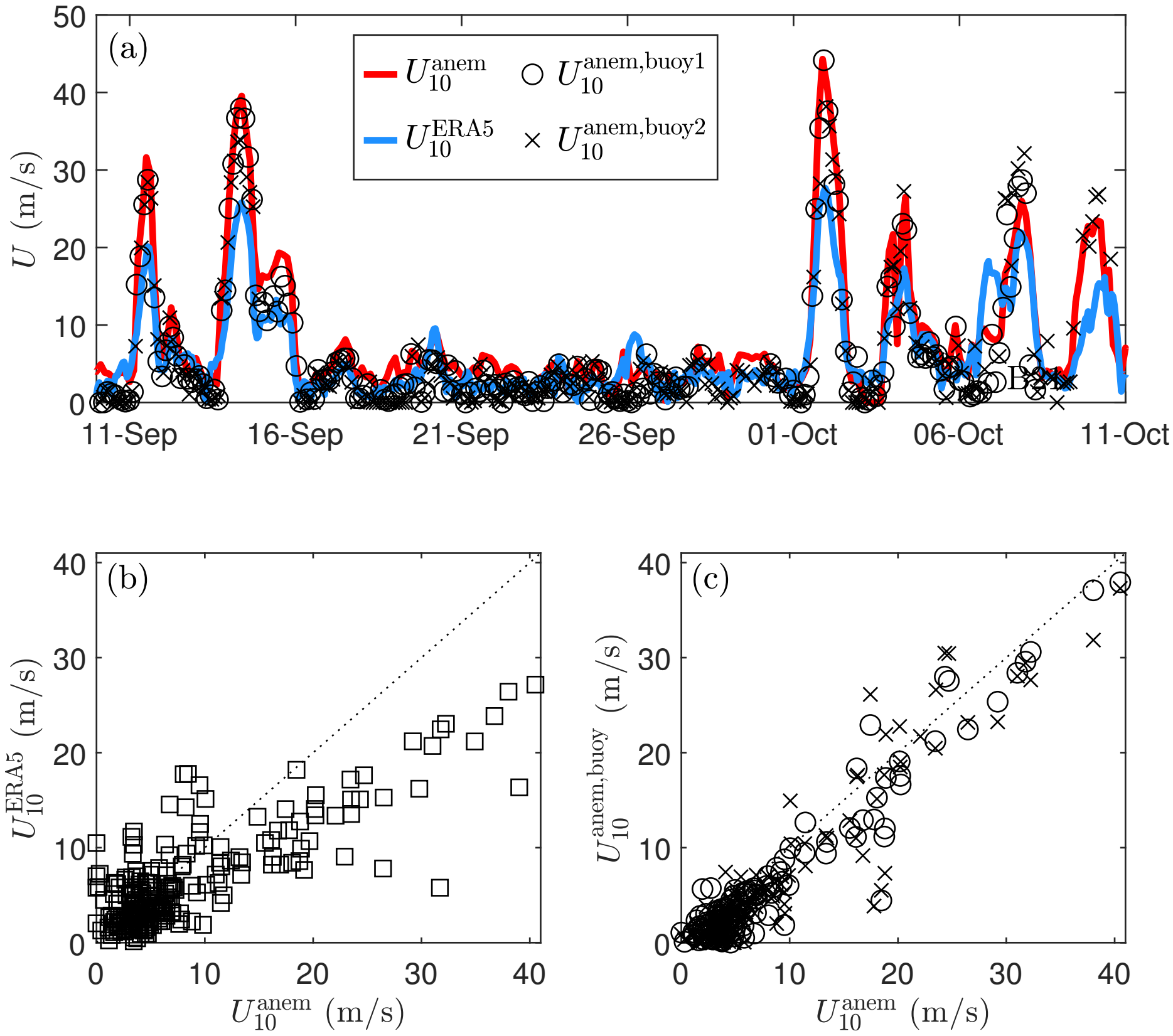

Fig. S14: Comparison between ERA5 wind speed (U_{10}^{ERA5}) and in-situ anemometer measurements at Casey Station (U_{10}^{anem}), and anemometer measurements from two buoys deployed on fast ice ($U_{10}^{\mathrm{anem,buoy}}$) during the 2021 field experiment.

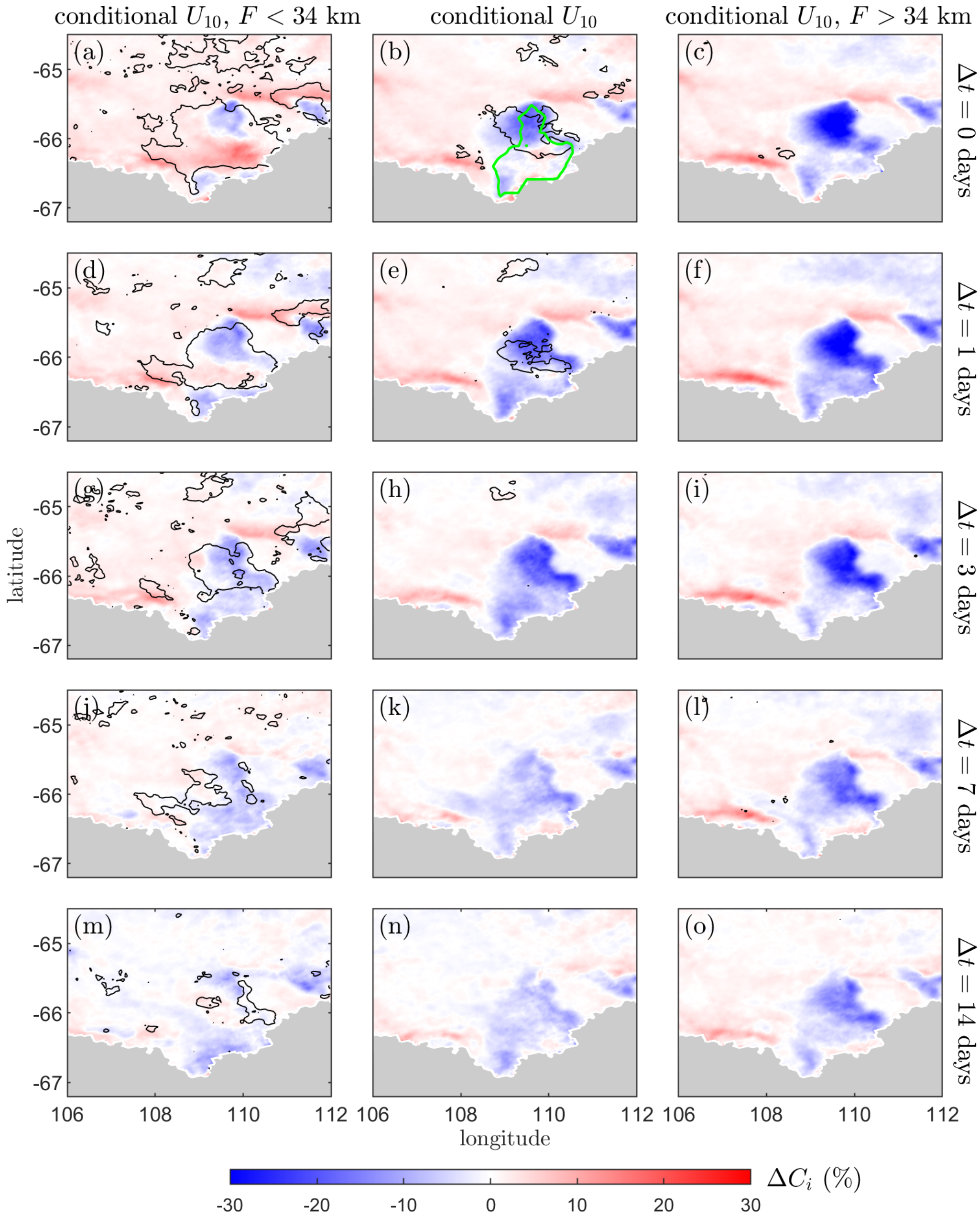

Fig. S15: Conditionally-averaged sea ice concentration difference maps for August–October, relative to three days before the samples. All columns are based on extreme-wind events. The middle column shows all extreme-wind cases, defined using the 95th percentile of U_{10}. The short-fetch (left column) and long-fetch (right column) wind-conditioned subsets were derived by selecting events exceeding the 90th percentile of U_{10} and then splitting these events by the median fetch length ($F = 34$ km). The left column corresponds to the right column in Fig. 8 of the main manuscript. Rows represent the lag time Δt relative to the sampled event. Black contours identify $p < 0.05$ relative to the extreme-I_{br} subset. The green contour in (b) identifies the region at which the polynya was present for at least 10% of the extreme-I_{br} subset.

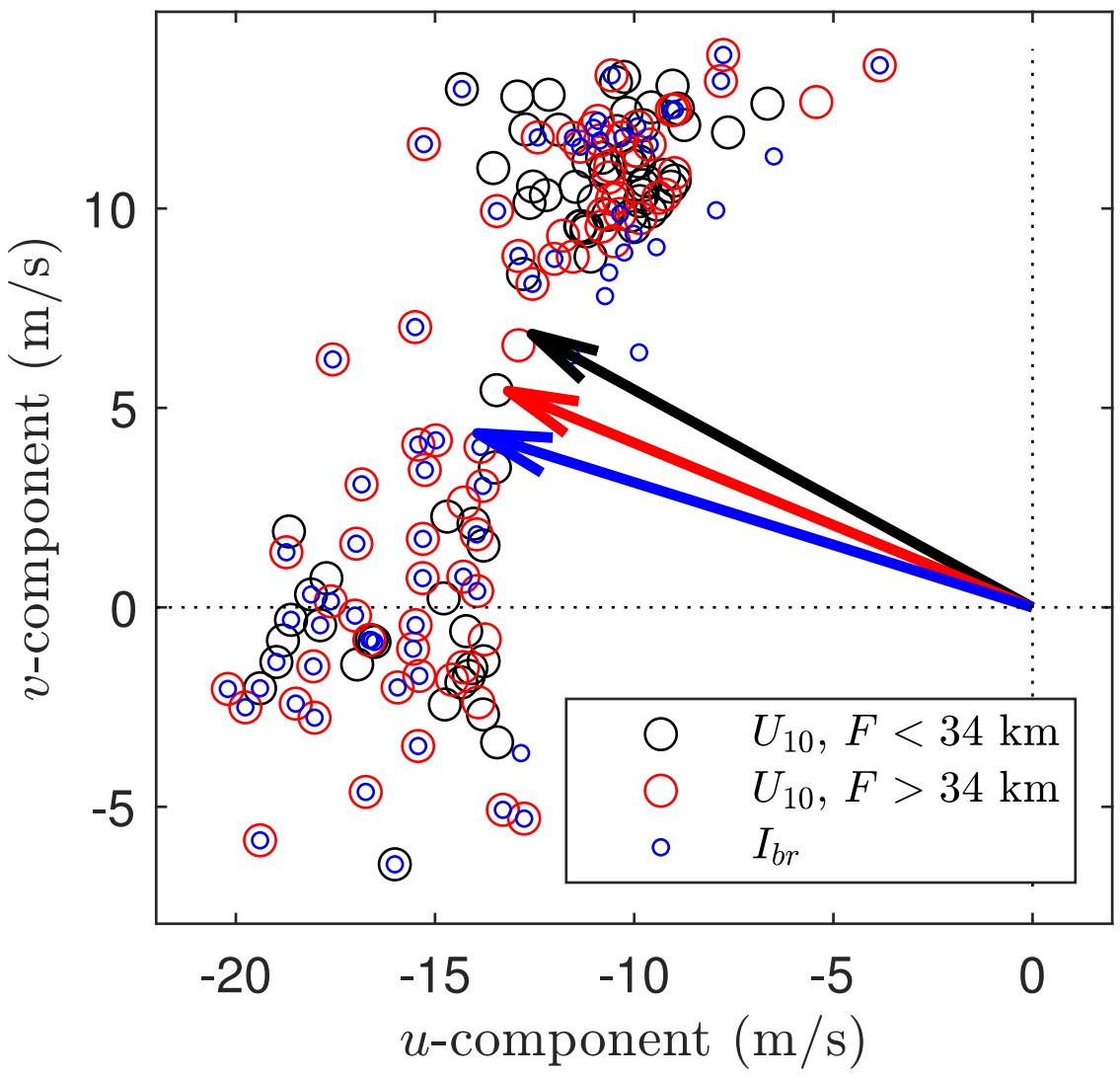

Fig. S16: Distribution of ERA5 u, v-wind components corresponding to the extreme values of I_{br} and U_{10}, with those of U_{10} split by short ($F < 34$ km) and long fetches ($F > 34$ km). The mean wind speed and direction for the subsets of short fetch, long fetch and I_{br} are 15.7, 15.7, and 16.1 m/s, and 118.6°, 112.4° and 107.4°, respectively.

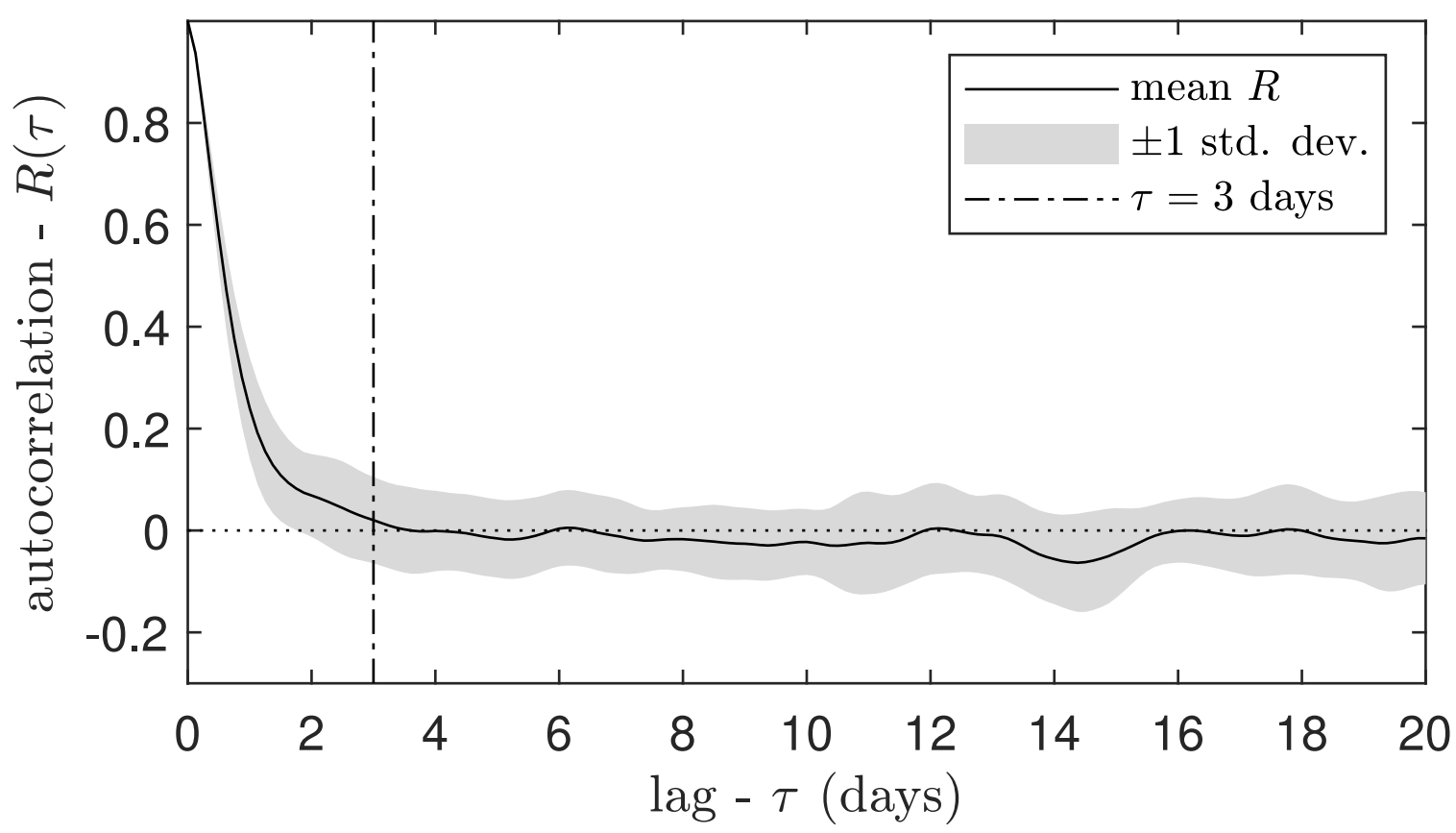

Fig. S17: Temporal autocorrelation of ERA5 10-m wind speeds across the Vincennes Bay Polynya (65–66° S, 108–109° E) during 2003–2022. The solid line shows the mean autocorrelation $R(\tau)$ and the shaded area the 5th–95th-percentile range across all grid cells. The effective decorrelation time, defined as the first zero-crossing, occurs at $\tau \approx 3.3$ days (vertical dash-dotted line), which is adopted as the reference time scale $t_{\mathrm{ref}} = 3$ days used in this study.

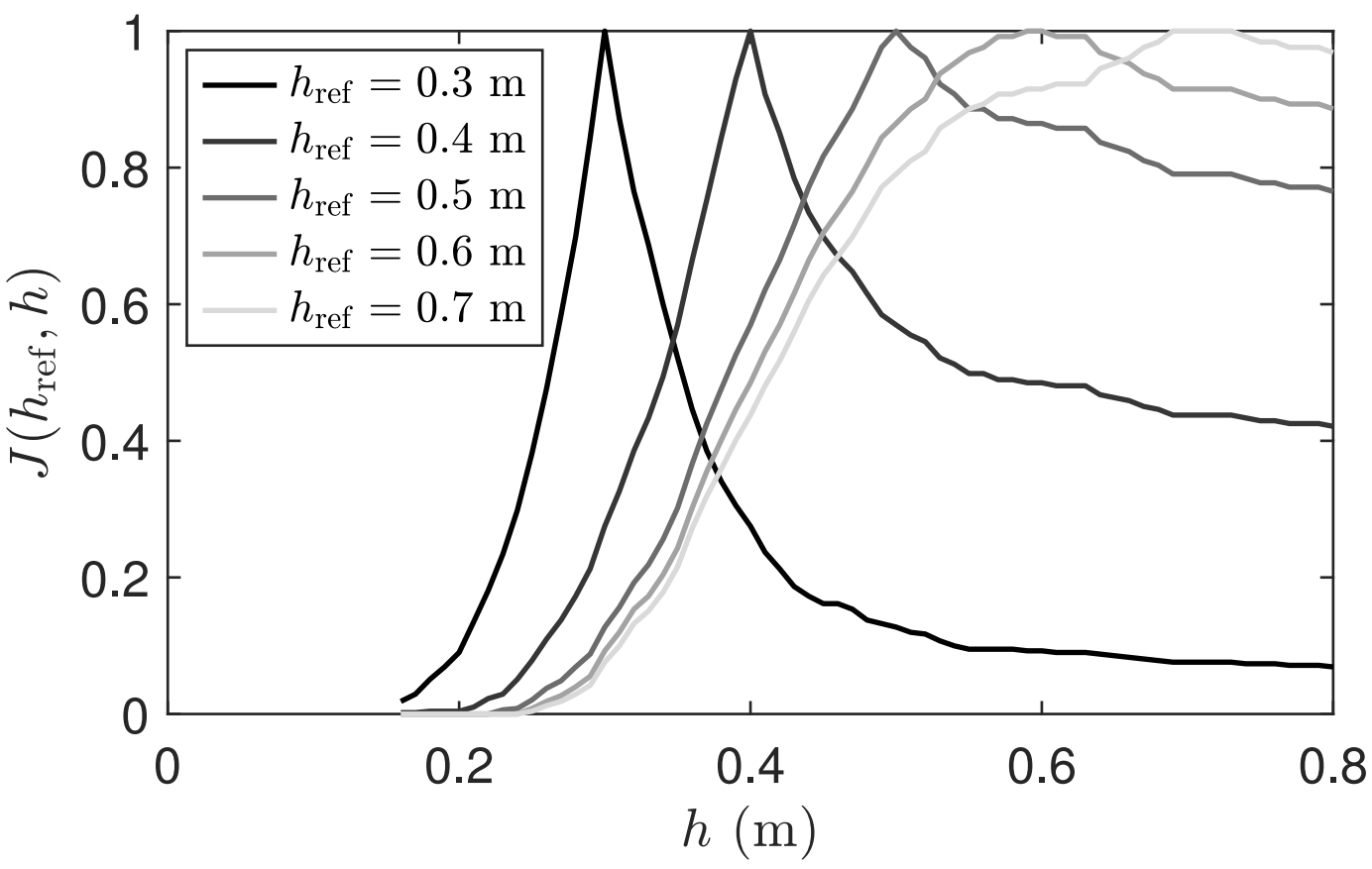

Fig. S18: The Jaccard index $J(h, h_{\rm ref})$ quantifies the overlap between subsets of extreme events identified using different ice-thickness values h and a fixed reference thickness $h_{\rm ref}$. Results are shown for $h_{\rm ref} = 0.3$, 0.4, 0.5, 0.6, and 0.7 m.

References

[1] Meylan, M.H., Bennetts, L.G., Kohout, A.L.: In situ measurements and analysis of ocean waves in the Antarctic marginal ice zone. Geophysical Research Letters **41**(14), 5046–5051 (2014) https://doi.org/10.1002/2014GL060809

[2] Voermans, J.J., Liu, Q., Marchenko, A., Rabault, J., Filchuk, K., Ryzhov, I., Heil, P., Waseda, T., Nose, T., Kodaira, T., *et al.*: Wave dispersion and dissipation in landfast ice: Comparison of observations against models. The Cryosphere **15**(12), 5557–5575 (2021) https://doi.org/10.5194/tc-15-5557-2021
