# Supplementary material for: Inside-outward breakup of sea ice by locally generated waves in the Vincennes Bay Polynya
Source: Nat Commun. 2026 Sep 29;17:11224. doi: 10.1038/s41467-026-77979-y (PMC13720198; doi:10.1038/s41467-026-77979-y)
Supplement: Supplementary file 2 [file 41467_2026_77979_MOESM2_ESM.pdf]

nature portfolio

Peer Review File

Inside-outward breakup of sea ice by locally generated waves in the Vincennes Bay Polynya

Corresponding Author: Dr Joey Voermans

This file contains all reviewer reports in order by version, followed by all author rebuttals in order by version.

Version 0:

Reviewer comments:

Reviewer #1

(Remarks to the Author)
This paper explores the importance of ocean surface waves in polynya evolution, a largely unexplored topic despite decades of sustained interest in coastal polynyas. After presenting a rare set of in situ wave observations, the authors apply fetch-limited wave growth relationships to satellite and reanalysis data to explore the potential for waves to fracture ice at the polynya edge. While the in situ observations don't reveal anything particularly novel about wave properties in polynyas, the finding that locally-generated waves frequently exceed the theoretical threshold for ice breaking is a novel and creative contribution of this work. The authors also hypothesize that wave-induced ice breakup results in sustained reductions in sea ice concentration in the polynya. While this is an important question, the conditional averaging approach used to demonstrate this result may not effectively disentangle wind effects and wave effects.

Specific comments:

What was the time period for which the fetch-limited relations were applied to the AMSR-derived polynya boundaries? The time range is listed as 2012-2022 on lines 32, 264, and 315 but as 2003-2022 in the Figure 6 caption and line 489.

Line 32-33: Perhaps it would be more accurate to say wave breakup enhances rather than "causes" sea ice concentration reductions, as this trend is present for the extreme wind events as well

Line 72: While I agree that wave observations are very sparse in polynyas, it should be noted that moored uplooking ADCPs can also be used to observe waves in areas with intermittent ice cover. For example, Hošeková et al. (2021 https://doi.org/10.1029/2021GL095103) observed several mid-winter wave events likely in coastal polynyas using five-beam ADCPs.

Section 2.1: I'm not sure what the WWIII simulations add to the study. It seems the primary motivation for including these simulations was to demonstrate that the observed waves are locally generated and not propagating swell (lines 115-121) but given the WWIII overestimation of swell relative to the buoys (lines 144-146) it does not seem like the WWIII results are trustworthy for this purpose. I find the wave age calculations, SAR data, and distance to the ice edge sufficient evidence that the waves are locally generated.

Line 237: The statement that "the observed waves follow the fetch-limited growth relations well" should be modified to indicate this is only true when the observations are collected outside of the ice. The observed waves do not follow the fetch-limited growth relations well for B21 and B22 due to wave attenuation in ice (lines 196-197).

Line 283: How much overlap is there in events identified using the extreme U10 and Ibr criteria? It seems likely that many extreme Ibr events occur under extreme wind conditions. If the goal is to disentangle the effects of waves from the effects of strong winds, perhaps the conditional averaging approach could be applied to strong wind events with short fetch vs. strong wind events with long fetch. This would help reduce overlap between the two forcing mechanisms

Are there trends over time in the large dataset of expected Hs and Ibr shown in Figure 6? Do you expect there might be trends over time in ice thickness that would influence the calculated value of Ibr?

Lines 324-325: It would be helpful to engage more deeply with the existing literature to discuss how waves promote advection/divergence of the ice cover through increased floe mobility or wave radiation stress. While I think the scoping choice to focus this study on the potential of waves to fracture ice is appropriate, it is likely that an alternative or additional impact of the momentum released by wave attenuation at the downwind end of the polynya is ice drift driven by wave radiation stress divergence. This process is discussed in Dai et al. (2019 https://doi.org/10.1016/j.ocemod.2018.11.006) and Thomson et al. (2021 https://doi.org/10.1029/2020GL090735).

In general, an early map figure with an overview of the polynya's location would be helpful for interpreting statements like "ice export is strongly constrained by the consolidated tongue of landfast ice extending northward" (Lines 325-326). A wind rose for the months used in the fetch-limited wave growth dataset would also be a helpful addition to such an overview figure.

Please provide more information about the wave buoy observations:
-Why do the buoys lack directional measurements?
-What is the noise floor?
-Do you expect the buoys remained embedded in large floes following breakout? How does this impact the buoys' ability to measure the open water conditions surrounding the floes into which they are embedded?
-Does using breakouts to "deploy" buoys bias the in situ observations towards particularly strong wind events (as strong forcing may be required to cause the breakouts)? This would not diminish the value of the in situ observations, but is worth noting if true. What was the numeric value of U10, 95 and how do the peak wind speeds shown in Figure S1 compare to that value?

Line 465: Is the Hs used to calculate Ibr the largest daily expected Hs modeled with the fetch-limited growth relations?

Line 493: Do you use daily-averaged ERA5 wind in these calculations to match the temporal frequency of the AMSR-derived polynya boundary dataset?

Lines 532-534: What was the distribution of peak periods calculated using the fetch-limited growth relations? In other words, please provide information for wave period similar to that reported for wave height in lines 246-247. I expect these locally-generated wind waves to have relatively short periods, so I am not sure I agree with the choice to focus on weakly-attenuating, longer period waves (line 538). What evidence is there for the statement that ice located 50-60 km from the ice edge is "well within reach of the most energetic waves generated in the polynya" (lines 542-543)? Even the 2 m waves (95th percentile Hs of the total dataset, line 247) observed during B24 appear to have periods of about 5 seconds (Figure 9); how far would you expect these waves to propagate into the ice pack?

Reviewer #2

(Remarks to the Author)
This is a review of the manuscript entitled "Inside-outward break-up of sea ice by locally generated waves in the Vincennes Bay Polynya" submitted to Nature Communications.

This study focuses on wave generation in an Antarctic polynya. It relies strongly on an observational dataset from wave buoys, initially deployed on landfast ice and which eventually crossed the polynya following a breakout event. These "opportunistic" measurements are highly valuable, as deploying buoys in a polynya is very challenging. Because the waves recorded by these buoys are large enough to likely impact the ice, the authors investigate:

• The origin of these waves: are they locally generated?
• Whether they can break the sea ice, thereby affecting its dynamical and thermodynamical properties.
• The evolution of sea ice around the polynya following intense wave events: is there an impact on sea-ice concentration that differs from "normal" conditions?

To address these questions, the authors complement their in situ observations with a wave model, a simple empirical model for fetch-limited wave growth, and various satellite imagery. They show that the waves observed by the buoys are locally generated and that they can exert sufficient strain to break the ice at the edge of the polynya. Using the fetch law together with winds from reanalysis, they further demonstrate that the wave events most likely to break the ice are associated with reductions in sea-ice area at the polynya edge. They also show that this reduction exhibits different patterns from those associated with extreme wind-speed events, suggesting that waves play a distinct role in reducing sea-ice cover, likely through their impact on sea-ice dynamics and mechanics.
The study is very thorough, the methods are appropriate, and the results support the conclusions. The findings are clearly novel and strongly benefit from the unique buoy dataset. Although waves have been reported in polynyas and it has been suggested that they play a role in their dynamics, this is, to my knowledge, the first study to provide repeated measurements of waves in the same polynyas together with a careful demonstration of their impact on sea ice. While the demonstration that locally-generated waves can break the ice at the polynya edge remains indirect, it is careful and convincing, and the observed wave properties leave little doubt that this process occurs. Regarding sea-ice concentration, I appreciated that the authors distinguish between the effects of waves and wind following extreme events.
I think the novelty of the findings and the thoroughness of the study warrant publication of this study after minor revisions.

L124: The periods are underestimated, but the wave heights are overestimated, correct?

Section 2.1: I found that the logical structure of some paragraphs was not always clear. I recommend starting each paragraph with a sentence that explicitly states its purpose in relation to the section's goal (distinguishing between wind waves and swell). For example, instead of "To further examine the discrepancies… (L135)", something like: "To further ensure that the observed waves are locally generated, we now examine the wave spectra surrounding…". This would help the reader to connect the information presented across the different figures.

The manuscript is generally clear and well written, but I found the discussion of the impact of sea ice on fetch-limited wave growth (L211–230) confusing. Figure 5 is not easy to interpret, and the motivation for using two attenuation rates is not entirely clear to me. I would suggest expanding the discussion of how to interpret the convergence/divergence of the two distances they compute. The sentence starting with "Notably (L226)" should also be clarified: why is it a "good" thing that Δx agrees with Δx_Hs and Δx_fp?

L228: Should this be 17 October instead of 12 October? If not, I am confused.

Section 2.3:
I appreciated the distinction between Ibr and U10. However, assessing the impact of wind speed is not exactly the same as assessing the influence of the wind more generally. The direction that maximises the fetch could also favour the opening of the polynya for reasons independent of waves (for instance, a lower resistance of sea ice to deformation in certain directions). Perhaps the authors could comment on this?

Discussion:

I think the authors could further emphasise the importance and implications of their results, particularly the significance of locally generated wind waves in polynyas compared to swell propagating from the open ocean in the MIZ. For instance, wave radiation stress is only briefly mentioned, although these short waves carry more momentum than swell. The results suggest this mechanism could significantly affect polynya opening rates by transferring momentum accumulated over the fetch to the ice edge. Stopa et al. (2018) showed that this effect can be comparable to wind stress in the outer MIZ, and it may similarly influence polynya opening rates (which could even be estimated with a simple back-of-the-envelope calculation assuming a given attenuation rate). It could well be as important a the weakening of the ice, which is much harder to estimate.

References:

Stopa, J. E., Sutherland, P., & Ardhuin, F. (2018). Strong and highly variable push of ocean waves on Southern Ocean sea ice. Proceedings of the National Academy of Sciences of the United States of America, 115(23), 5861–5865. https://doi.org/10.1073/pnas.1802011115

Version 1:

Reviewer comments:

Reviewer #1

(Remarks to the Author)
This paper makes a strong argument that waves are a central part of polynya dynamics and that the conventional understanding of polynyas as primary wind-driven overlooks waves as important sources of energy and momentum for the polynya-edge ice. Apart from some minor comments listed below, my major concerns have been addressed. I recommend the paper for publication.

L23: It is unnecessarily restrictive to say Antarctic sea ice (there are also coastal polynyas in the Arctic)

L74: This is a small quibble, but it is certainly possible to measure ocean surface waves in ice impacted areas using deep water moorings. For example, this is done routinely in the Arctic by the Beaufort Gyre Exploration Project moorings (i.e., see Thomson and Rogers 2014, https://doi.org/10.1002/2014GL059983)

Figure 1 is a very nice addition to the paper, thank you

Figure 2: What is the difference between the black and gray scatter points?

L105: Fix typo fst -> fast

L109: "satisfied throughout the deployments": This does not seem to be strictly true in Figure S1. Perhaps you mean it is satisfied while the buoys are in open water crossing the polynya?

L145: Might be helpful to mark the noise floor on the spectra in Fig 4 or state its expected magnitude in the caption.

L230: The need to daily average the wind forcing is another important assumption/source of uncertainty to note as it smooths

the high frequency gusts which would be expected to contribute disproportionately to wave growth

L273: Perhaps better to say “wave forcing” than “wave-induced ice break-up” here - you have shown that the strong wave conditions are associated with sea ice reduction, not that the break up (rather than, for example, wave-induced ice transport) caused the ice reduction

L276: “A similar analysis…” Based on the S14 figure caption, it does not appear that the updated conditional averaging approach (using short fetch distances) has been applied for these ice thickness maps (or for S15).

L280: I think Fig S14 is cited out of order (S11 and S12 have not been cited yet, and S13 may not be cited at all)

L315: “further supporting the interpretation of wave-driven localized deformation and thickening” - Sutherland and Dumont 2018 (10.1175/JPO-D-17-0167.1) is a good reference here

L332-334: The estimates of wave radiation stress are a great addition to the paper. However, I am struggling to reconcile the finding that that wave radiation stress impacts are confined to ~1 km from the ice edge with the argument that, from a fracture perspective, “waves may remain dynamically relevant over distances of approximately 10–80 km into the pack” (lines 583-584). I understand that these are different processes and that wave radiation stress convergence depends on the attenuation rate while l_br depends on the wave height, but it still seems like there should be a contradiction here. If waves attenuate rapidly enough to impart a strong wave radiation stress convergence in the first ~1 km of the ice pack, how is it that they remain large enough to break the ice more than ten times that distance into the ice pack? I can imagine that it’s possible for both of these things to be true, but it would be helpful if the text in lines 334-338 (“We therefore interpret…”) were more specific about this interplay.

L367: The additional information about the buoys is appreciated

L523: I think this may be the incorrect reference for this methodology (also for the reference on line 330)? I think you actually mean this (other) Stopa et al. 2018 paper: https://doi.org/10.1073/pnas.1802011115

Fig S12: In the caption, please explain the thin line width and bold lines. Is this a subset of all polynya events and their mean?

Reviewer #2

(Remarks to the Author)
This is my second review of the manuscript entitled “Inside-outward break-up of sea ice by locally generated waves in the Vincennes Bay Polynya,” submitted to Nature Communications.

As noted in my first review, the study is thorough, the methods are appropriate, and the results support the conclusions. The authors have addressed my comments very well. In particular, Section 2 is now much easier to read, as the purpose of each tool and diagnostic is presented more clearly. The additional work on conditional averaging, together with the discussion of wave radiation stress, also strengthens the authors’ argument that waves contribute to the redistribution of sea ice along the margins of the polynya. I therefore support the publication of this manuscript in Nature Communications.

typo:
L105--> fast ice

Open Access This Peer Review File is licensed under a Creative Commons Attribution 4.0 International License, which permits use, sharing, adaptation, distribution and reproduction in any medium or format, as long as you give appropriate credit to the original author(s) and the source, provide a link to the Creative Commons license, and indicate if changes were made.
In cases where reviewers are anonymous, credit should be given to 'Anonymous Referee' and the source.
The images or other third party material in this Peer Review File are included in the article's Creative Commons license, unless indicated otherwise in a credit line to the material. If material is not included in the article's Creative Commons license and your intended use is not permitted by statutory regulation or exceeds the permitted use, you will need to obtain permission directly from the copyright holder.
To view a copy of this license, visit https://creativecommons.org/licenses/by/4.0/

Response to Reviewer 1

Manuscript title: *Inside-outward break-up of sea ice by locally generated waves in the Vincennes Bay Polynya*

We thank the reviewer for their careful reading of the manuscript and for the constructive comments. We believe these comments have helped improve the clarity and strength of the manuscript. The main revisions are summarized below:

(1) We have more clearly disentangled the effects of wind and waves in the conditional averaging analysis by introducing a high-wind, short-fetch comparison. This provides a more direct assessment of the contribution of wave-induced ice break-up beyond wind forcing alone and strengthens the support for our conclusions.
(2) We have extended the conditional averaging analysis of sea-ice concentration from 2012–2022 to 2003–2022 by incorporating AMSR-E observations. The extended record yields consistent results while improving the statistical robustness of the analysis.
(3) We have expanded the discussion of the physical mechanisms linking wave-induced break-up to the observed sea-ice response, including the complementary role of the wave radiation stress.

Detailed responses to each comment are provided below.

Reviewer #1
This paper explores the importance of ocean surface waves in polynya evolution, a largely unexplored topic despite decades of sustained interest in coastal polynyas. After presenting a rare set of in situ wave observations, the authors apply fetch-limited wave growth relationships to satellite and reanalysis data to explore the potential for waves to fracture ice at the polynya edge. While the in situ observations don't reveal anything particularly novel about wave properties in polynyas, the finding that locally-generated waves frequently exceed the theoretical threshold for ice breaking is a novel and creative contribution of this work. The authors also hypothesize that wave-induced ice breakup results in sustained reductions in sea ice concentration in the polynya. While this is an important question, the conditional averaging approach used to demonstrate this result may not effectively disentangle wind effects and wave effects.

Specific comments:

What was the time period for which the fetch-limited relations were applied to the AMSR-derived polynya boundaries? The time range is listed as 2012-2022 on lines 32, 264, and 315 but as 2003-2022 in the Figure 6 caption and line 489.

We thank the reviewer for drawing attention to the different analysis periods reported in the manuscript. The polynya-edge dataset of Lin et al. combines AMSR-E and AMSR2 observations and spans 2003-2022. In contrast, the original conditional-averaging analysis in our study used AMSR2 sea-ice concentration data only and therefore covered 2012-2022.

For consistency, we have now extended the conditional-averaging analysis to include AMSR-E observations, so that both the polynya-edge and sea-ice concentration datasets cover 2003-2022. The inclusion of AMSR-E increases the sample size and strengthens the statistical significance of the analysis, further supporting the robustness of the results. We have revised the relevant text and figure captions accordingly.

Line 32-33: Perhaps it would be more accurate to say wave breakup enhances rather than “causes” sea ice concentration reductions, as this trend is present for the extreme wind events as well

We agree with the reviewer. We have changed ‘causes’ to ‘associated with’ (line 32, revised manuscript).

Line 72: While I agree that wave observations are very sparse in polynyas, it should be noted that moored uplooking ADCPs can also be used to observe waves in areas with intermittent ice cover. For example, Hošeková et al. (2021 https://doi.org/10.1029/2021GL095103) observed several mid-winter wave events likely in coastal polynyas using five-beam ADCPs.

We agree with the reviewer that subsurface instrumentation may provide observations of waves as well. We have added the following to the Introduction at lines 72-74:

“Moored upward-looking acoustic Doppler current profilers provide a viable alternative for measuring waves from beneath the surface [14], although their application in Antarctic polynyas may be constrained by water depth.”

Section 2.1: I’m not sure what the WWIII simulations add to the study. It seems the primary motivation for including these simulations was to demonstrate that the observed waves are locally generated and not propagating swell (lines 115-121) but given the WWIII overestimation of swell relative to the buoys (lines 144-146) it does not seem like the WWIII results are trustworthy for this purpose. I find the wave age calculations, SAR data, and distance to the ice edge sufficient evidence that the waves are locally generated.

We agree that the WWIII simulations struggle to reproduce the finer details of the buoy observations. Nevertheless, we decided to retain them because they provide useful regional context for interpreting the measurements, particularly the observed spectral components and the influence of wind and sea-ice conditions. We acknowledge that this purpose was not sufficiently clear in the original manuscript. In the revised version, we have therefore reduced the amount of text devoted to the simulations, and restructured the section so that the WWIII results support the interpretation of the observations rather than serve as proof of local wave generation. We refer the reviewer to Section 2.1 of the revised manuscript.

Line 237: The statement that “the observed waves follow the fetch-limited growth relations well” should be modified to indicate this is only true when the observations are collected outside of the ice. The observed waves do not follow the fetch-limited growth relations well for B21 and B22 due to wave attenuation in ice (lines 196-197).

Indeed, this only applied to observations made within the polynya. We have rephrased this sentence to (lines 213-214, revised manuscript):

“Since the observed waves within the polynya follow the fetch-limited growth relations well …”

Line 283: How much overlap is there in events identified using the extreme U10 and Ibr criteria? It seems likely that many extreme Ibr events occur under extreme wind conditions. If the goal is to disentangle the effects of waves from the effects of strong winds, perhaps the conditional averaging approach could be applied to strong wind events with short fetch vs.

strong wind events with long fetch. This would help reduce overlap between the two forcing mechanisms

We thank the reviewer for this suggestion. The reviewer is correct that there is substantial overlap between the extremes in I_{br} and U_{10} (about 50%), as large values of I_{br} tend to occur under strong winds together with sufficiently long fetches. We therefore agree that comparing strong-wind events under short- and long-fetch conditions provides a more effective means of disentangling the effects of waves from that of strong wind forcing alone.

Following this suggestion, we selected events with wind speeds above the 90th percentile and divided these into short- and long-fetch subsets using the median fetch (34 km), resulting in subsets of comparable size to the 95th-percentile I_{br} selection. We note that the overlap between the high-wind, short-fetch subset and the extreme-I_{br} subset is only 7%. As presented in Figure 8 (revised manuscript), the differences in the conditionally averaged changes in sea ice concentration between extremes in I_{br} and winds with short fetches are considerable and now also statistically significant across large spatial areas.

We have also included an additional figure in the Supplementary Information (Figure S13), comparing the short fetch, long fetch, and the original extreme wind subset, defined by its 95th percentile. We would like to refer the reviewer to Figure 8 and Figure S13 of the revised manuscript and revised Supplementary Information, respectively.

Are there trends over time in the large dataset of expected Hs and Ibr shown in Figure 6? Do you expect there might be trends over time in ice thickness that would influence the calculated value of Ibr?

We tested for temporal trends in expected H_s and I_{br} by calculating the median value for August-October in each year and fitting a linear trend to these annual medians. We found no statistically significant evidence of a linear trend in either expected H_s ($p = 0.66$) or I_{br} ($p = 0.42$) over 2003–2022 (see Figure below). Detecting long-term trends in this region may be complicated by the strong spatial and temporal variability of the polynya and surrounding ice pack.

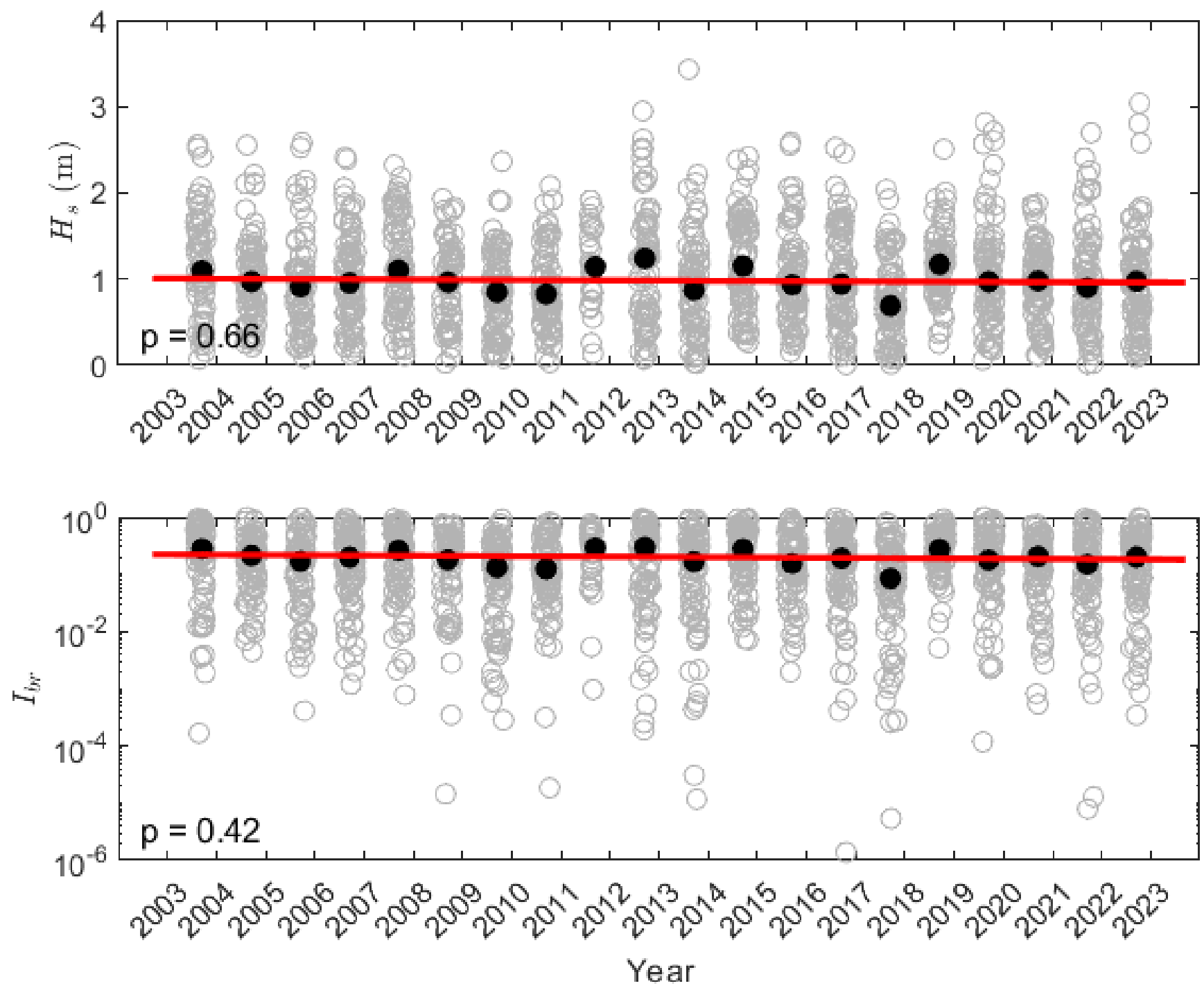

Trends in sea-ice thickness could in principle influence I_{br}, as it scales roughly linearly with thickness. Sensitivity to plausible centimetre-scale thickness changes over one or two decades is small compared with the event-to-event variability in wave height, wavelength and ice conditions. We therefore do not expect any plausible thickness trend over the study period to materially affect the interpretation of our results.

Lines 324-325: It would be helpful to engage more deeply with the existing literature to discuss how waves promote advection/divergence of the ice cover through increased floe mobility or wave radiation stress. While I think the scoping choice to focus this study on the potential of waves to fracture ice is appropriate, it is likely that an alternative or additional impact of the momentum released by wave attenuation at the downwind end of the polynya is ice drift driven by wave radiation stress divergence. This process is discussed in Dai et al. (2019 https://doi.org/10.1016/j.ocemod.2018.11.006) and Thomson et al. (2021 https://doi.org/10.1029/2020GL090735).

We thank the reviewer for this suggestion. We have included estimates of the on-ice wave-induced stress resulting from wave attenuation, presented in the revised Supplementary Information (Fig. S12), and expanded the Discussion accordingly. We would like to refer the reviewer to the Discussion at lines 321-338, which now reads:

"The inferred thickening and positive concentration anomalies cannot be explained by wave-induced break-up alone, but indicate redistribution and compaction of the ice cover under convergent forcing. In this context, wave radiation stress provides a physically linked momentum source: as locally generated waves attenuate, their momentum is transferred to the ice through convergence of the wave radiation stress, which can drive ice drift, compaction, rafting and ice-edge deformation [28, 29, 31, 32]. Our scaling estimates (Section 4.6) suggest that this wave-induced stress can be comparable to, or locally exceed, the wind stress close to the polynya ice edge, particularly under high-attenuation conditions (Suppl. Fig. S12). The estimated stress is also larger than typical estimates for longer-period swell in the outer MIZ [29], reflecting the stronger momentum convergence associated with the rapid attenuation of short, locally generated waves. However, this same rapid attenuation confines the direct forcing to a relatively narrow band of the order of a kilometer around the polynya edge [32]. We therefore interpret the observed response as an interplay between wave-induced break-up, radiation-stress forcing and wind-driven redistribution: break-up preconditions the ice cover for redistribution by reducing its mechanical coherence and producing smaller, more mobile floes, while wind and wave-induced stresses provide the convergent forcing needed to advect, compact and raft the fragmented ice [28, 33]."

In general, an early map figure with an overview of the polynya's location would be helpful for interpreting statements like "ice export is strongly constrained by the consolidated tongue of landfast ice extending northward" (Lines 325-326). A wind rose for the months used in the fetch-limited wave growth dataset would also be a helpful addition to such an overview figure.

We thank the reviewer for the suggestion. We have now added an overview figure of the polynya, and have included a wind rose as well, see Fig. 1 in revised manuscript.

Please provide more information about the wave buoy observations:
-Why do the buoys lack directional measurements?

-What is the noise floor?
-Do you expect the buoys remained embedded in large floes following breakout? How does this impact the buoys' ability to measure the open water conditions surrounding the floes into which they are embedded?
-Does using breakouts to "deploy" buoys bias the in situ observations towards particularly strong wind events (as strong forcing may be required to cause the breakouts)? This would not diminish the value of the in situ observations, but is worth noting if true. What was the numeric value of U10, 95 and how do the peak wind speeds shown in Figure S1 compare to that value?

The buoys measure one-dimensional wave spectra only and do not provide directional information. Directional estimates from compact wave buoys commonly rely on horizontal GPS-derived velocities, but these may become unreliable in sea ice because horizontal motion can be constrained by the ice. Magnetometer-based directional estimates are also problematic in polar regions because of the weak horizontal magnetic-field component and potential interference from batteries and electronics within compact instruments.

While the accelerometer noise density is $75\mu g/\sqrt{Hz}$, the effective noise floor of the processed elevation spectra also depends on the gyroscope, sensor fusion, and the local wave conditions. It is therefore more appropriately identified from the background support level of the observed spectra than from the accelerometer specification alone. Previous validation of these buoy designs indicates that they can resolve centimetre-scale wave motions and, under favourable conditions, millimetre-scale motions (Rabault et al., 2020; Rabault et al. 2022). The wave-energy levels considered in the present study are well above these levels.

The 90th and 95th percentiles of U_{10} are 13.6 and 15.2 m/s, respectively (we note that in reality this is likely to be higher, as ERA5 tends to underestimate winds at the site of interest). We have included these values now at lines 537 and 541 of the revised manuscript.

ERA5 winds during all buoy breakouts exceeded the 95th percentile. In fact, both the 2021 and the 2022 crossings are included in the extreme I_{br} subset (the polynya edge dataset goes until 2022, therefore 2024 and 2025 breakouts are not included). This indeed means that the wave observations presented are not a reflection of full range of wave conditions in the Vincennes Bay Polynya, instead, they are biased towards the extreme wind events.

We have made the following edits to make the reader aware of these details. At lines 244-246, the following was added:

"Notably, the breakout days of B21 and B22 both fall within the extreme-I_{br} subset, indicating that the in situ observations sampled the high-break-up-potential conditions targeted by the broader statistical analysis."

And at lines 367-379, the following paragraph was added:

The wave measurements were obtained using open-source waves-in-ice buoys [15, 16]. These instruments transmit GPS positions and one-dimensional wave energy spectra, $E(f)$, derived from the measured vertical motion of the ice. We therefore restrict the analysis to non-directional bulk wave parameters, including significant wave height and peak period. Previous validation of these buoys indicates that they can resolve centimetre-scale wave motions and, under favourable conditions,

millimetre-scale motions [15, 16]. Following breakout, the buoys likely remained embedded in drifting ice fragments or floes, so the measurements are interpreted as the wave field experienced by the ice rather than as fully open-water wave conditions. As such, they may provide conservative estimates of the wave energy that would develop under equivalent open-water conditions. Since the buoys entered the polynya only following breakout from fast ice, these observations preferentially sample strong-forcing conditions and should not be interpreted as a random sample of all polynya wave states.

Line 465: Is the Hs used to calculate Ibr the largest daily expected Hs modeled with the fetch-limited growth relations?

The reviewer is correct that the wave field characteristics are estimated based on the fetch-limited growth conditions. We refer the reviewer to line 501 of the revised manuscript.

Line 493: Do you use daily-averaged ERA5 wind in these calculations to match the temporal frequency of the AMSR-derived polynya boundary dataset?

The reviewer is correct, we use the daily average wind speed and direction. This detail was missing and has now been added at line 216 of the revised manuscript, and the Methods section at line 509.

Lines 532-534: What was the distribution of peak periods calculated using the fetch-limited growth relations? In other words, please provide information for wave period similar to that reported for wave height in lines 246-247. I expect these locally-generated wind waves to have relatively short periods, so I am not sure I agree with the choice to focus on weakly-attenuating, longer period waves (line 538). What evidence is there for the statement that ice located 50-60 km from the ice edge is “well within reach of the most energetic waves generated in the polynya” (lines 542-543)? Even the 2 m waves (95th percentile Hs of the total dataset, line 247) observed during B24 appear to have periods of about 5 seconds (Figure 9); how far would you expect these waves to propagate into the ice pack?

We agree with the reviewer that the locally generated waves considered here have relatively short peak periods compared with open-ocean swell. Across the full dataset, the 95th percentile of T_p is 5.6 s. For the subset of events in which I_{br} exceeds its 95th percentile, the mean peak period is approximately 6 s.

We acknowledge that our original description of these waves as weakly attenuating was unclear. We intended to describe their attenuation relative to that of the shorter, 3-4 s waves generated within the polynya on average, rather than relative to Southern Ocean swell. For example, the attenuation coefficient for a 6 s wave is approximately four to five times smaller than that for a 3.5 s wave under the parameterizations considered here. These relatively longer-period local waves therefore have greater potential to penetrate into the surrounding pack ice, although their spatial reach remains strongly dependent on the ice type and attenuation regime.

To estimate how far these waves may have an impact, we considered all events for which I_{br} exceeded its 95th percentile and estimated the decay of wave energy and I_{br} with distance into the ice using the low-attenuation parameterization of Meylan et al. (2014) and the high-attenuation parameterization of Voermans et al. (2021) (see figure below).

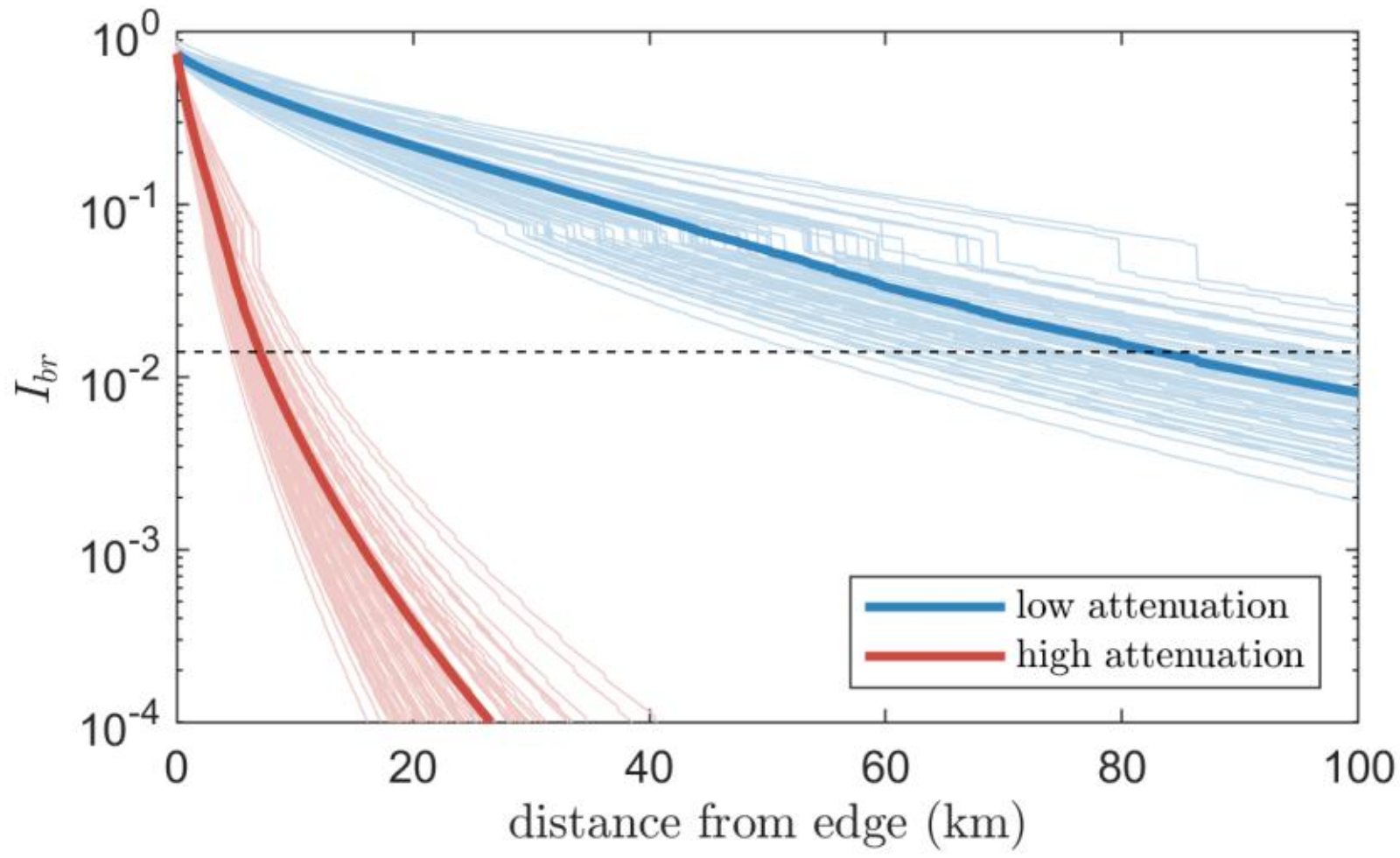

Under the low-attenuation case, I_{br} can remain above the critical threshold for distances exceeding 80 km beyond the polynya edge, whereas under the high-attenuation case this distance is reduced to approximately 10 km on average. We interpret these cases as approximate upper and lower bounds, respectively, on wave penetration under the ice conditions considered here.

We have revised the manuscript to clarify that the expected penetration distance depends on the attenuation regime and is estimated to range from approximately 10 to 80 km for the events considered here. The related paragraph at lines 574-584 now reads:

"To capture wave events with greater spatial impact, we therefore consider a higher, more representative ice thickness for such an area. Here we consider $h \approx 0.5$ m, for which the extremes correspond to longer-period waves of about 6 s. These waves attenuate less rapidly than shorter-period waves, penetrate farther into the pack, … . Taking $\alpha_H = 1.3 \times 10^{-1} f^{2.66}$ [18] and $\alpha_L = 2.12 \times 10^{-3} f^2 + 4.59 \times 10^{-2} f^4$ [41] as representative high- and low-attenuation bounds, respectively, we estimate that such waves may remain dynamically relevant over distances of approximately 10-80 km into the pack."

Response to Reviewer 2

Manuscript title: *Inside-outward break-up of sea ice by locally generated waves in the Vincennes Bay Polynya*

We thank the reviewer for their careful reading of the manuscript and for the constructive comments. We believe these comments have helped improve the clarity and strength of the manuscript. The main revisions are summarized below:

(1) We have more clearly disentangled the effects of wind and waves in the conditional averaging analysis by introducing a high-wind, short-fetch comparison. This provides a more direct assessment of the contribution of wave-induced ice break-up beyond wind forcing alone and strengthens the support for our conclusions.
(2) We have extended the conditional averaging analysis of sea-ice concentration from 2012–2022 to 2003–2022 by incorporating AMSR-E observations. The extended record yields consistent results while improving the statistical robustness of the analysis.
(3) We have expanded the discussion of the physical mechanisms linking wave-induced break-up to the observed sea-ice response, including the complementary role of the wave radiation stress.

Detailed responses to each comment are provided below.

Reviewer #2
This is a review of the manuscript entitled "Inside-outward break-up of sea ice by locally generated waves in the Vincennes Bay Polynya" submitted to Nature Communications.

This study focuses on wave generation in an Antarctic polynya. It relies strongly on an observational dataset from wave buoys, initially deployed on landfast ice and which eventually crossed the polynya following a breakout event. These “opportunistic” measurements are highly valuable, as deploying buoys in a polynya is very challenging. Because the waves recorded by these buoys are large enough to likely impact the ice, the authors investigate:

• The origin of these waves: are they locally generated?
• Whether they can break the sea ice, thereby affecting its dynamical and thermodynamical properties.
• The evolution of sea ice around the polynya following intense wave events: is there an impact on sea-ice concentration that differs from “normal” conditions?

To address these questions, the authors complement their in situ observations with a wave model, a simple empirical model for fetch-limited wave growth, and various satellite imagery. They show that the waves observed by the buoys are locally generated and that they can exert sufficient strain to break the ice at the edge of the polynya. Using the fetch law together with winds from reanalysis, they further demonstrate that the wave events most likely to break the ice are associated with reductions in sea-ice area at the polynya edge. They also show that this reduction exhibits different patterns from those associated with extreme wind-speed events, suggesting that waves play a distinct role in reducing sea-ice cover, likely through their impact on sea-ice dynamics and mechanics.

The study is very thorough, the methods are appropriate, and the results support the conclusions. The findings are clearly novel and strongly benefit from the unique buoy dataset. Although waves have been reported in polynyas and it has been suggested that they play a role in their dynamics, this is, to my knowledge, the first study to provide repeated measurements of waves in the same polynyas together with a careful demonstration of their impact on sea ice. While the demonstration that locally-generated waves can break the ice at the polynya edge remains indirect, it is careful and convincing, and the observed wave properties leave little doubt that this process occurs. Regarding sea-ice concentration, I appreciated that the authors distinguish between the effects of waves and wind following extreme events.
I think the novelty of the findings and the thoroughness of the study warrant publication of this study after minor revisions.

L124: The periods are underestimated, but the wave heights are overestimated, correct?

We thank the reviewer for noticing this. Indeed, the periods are underestimated and the wave heights overestimated. B25 is the exception to this, where both are underestimated. We have now corrected this in the manuscript, see lines 116-120 of revised manuscript.

Section 2.1: I found that the logical structure of some paragraphs was not always clear. I recommend starting each paragraph with a sentence that explicitly states its purpose in relation to the section's goal (distinguishing between wind waves and swell). For example, instead of "To further examine the discrepancies… (L135)", something like: "To further ensure that the observed waves are locally generated, we now examine the wave spectra surrounding…". This would help the reader to connect the information presented across the different figures.

We thank the reviewer for this suggestion. We have edited this section to improve clarity and would like to refer the reviewer to Section 2.1 of the revised manuscript.

The manuscript is generally clear and well written, but I found the discussion of the impact of sea ice on fetch-limited wave growth (L211–230) confusing. Figure 5 is not easy to interpret, and the motivation for using two attenuation rates is not entirely clear to me. I would suggest expanding the discussion of how to interpret the convergence/divergence of the two distances they compute. The sentence starting with "Notably (L226)" should also be clarified: why is it a "good" thing that Δx agrees with Δx_Hs and Δx_fp?

We acknowledge that this part of the manuscript was not sufficiently clear. The purpose of this analysis is to test whether the mismatch between the fetch-limited wave estimates and the buoy observations of B22 can be explained by wave attenuation in the sea ice present between the polynya ice edge and the buoy.

To make this comparison, we estimate the distance of sea ice that would be required to reduce the fetch-limited estimates to the observed values. This is done separately for significant wave height and peak frequency, giving the distances Δx_{Hs} and Δx_{fp}, respectively. If these two distances agree, it implies that the mismatch can be explained by wave-ice interactions over a common propagation distance through the sea ice. In other words, this supports the interpretation that the apparent deviation from fetch-limited growth is caused by attenuation in the sea ice, rather than by a failure of the fetch-limited framework itself.

The wave attenuation rate is not known a priori, which is why we use two different parameterizations: one representative of stronger attenuation in consolidated ice, and one representative of weaker attenuation in broken or loosely packed sea ice. We refer to these as α_H and α_L, respectively. They may be interpreted as approximate upper and lower bounds on the expected attenuation by sea ice. When compared with the actual distance between the polynya ice edge and the buoy, these estimates can also provide information on the type of ice experienced by the waves. For this, it is not necessary for Δx to agree with Δx_{Hs} and Δx_{fp}.

To improve the clarity of the analysis, we have edited the corresponding paragraph in Section 2.2 to now read:

To show that wave attenuation by sea ice can explain the anomalous apparent wave growth observed for B22, we first construct a JONSWAP spectrum using the significant wave height H_s^F and peak frequency f_p^F predicted from the open-water fetch-limited growth relations for the local wind and fetch conditions. We then apply two sea-ice attenuation regimes to the spectrum: a low rate (Eq. 3) representative of pancake ice, and a high rate ($\alpha_H = 1.3 \times 10^{-1} f^{2.66}$) representative of fast ice [18]. We then determine the effective distance Δx_{fp} required for the attenuated spectral peak to reproduce f_p^{obs} and, equivalently, the effective distance Δx_{Hs} required for the attenuated spectrum to reproduce H_s^{obs}. If these independently inferred distances are comparable, the observed reduction in wave energy and peak frequency can be explained by attenuation by sea-ice rather than by a departure from fetch-limited wave growth. Using this approach, we find that Δx_{Hs} and Δx_{fp} converge for times before 17 October 2022 and after 12:00 on 17 October 2022 (Fig. 6). This convergence, together with the satellite-derived Δx falling within the range predicted by the two attenuation scenarios, indicates that the deviations from fetch-limited growth can be readily explained by sea-ice attenuation. In contrast, during the early hours of 17 October 2022, Δx_{Hs} and Δx_{fp} diverge substantially, suggesting that attenuation alone cannot account for the observations. The strong drop in wind speed recorded by the land-based anemometer around this time likely played a role.

L228: Should this be 17 October instead of 12 October? If not, I am confused.

We thank the reviewer for noticing this error. This should indeed be 17 October.

Section 2.3:
I appreciated the distinction between Ibr and U10. However, assessing the impact of wind speed is not exactly the same as assessing the influence of the wind more generally. The direction that maximises the fetch could also favour the opening of the polynya for reasons independent of waves (for instance, a lower resistance of sea ice to deformation in certain directions). Perhaps the authors could comment on this?

The reviewer raises an important point. We agree that wind direction, and not only wind speed, may influence polynya opening independently of waves. For Vincennes Bay Polynya, the strongest wind events are predominantly easterly to southeasterly (also see the new Figure 1 for the wind rose). This is reflected also in the wind characteristics of the subsets of extreme I_{br} and U_{10}, which are very similar in both magnitude and direction. We have added Figure S11 in the Supplementary Information providing the wind characteristics of the different subsets. This suggests that, at least for this polynya, the comparison is not impacted by differences in wind direction.

Discussion:

I think the authors could further emphasise the importance and implications of their results, particularly the significance of locally generated wind waves in polynyas compared to swell propagating from the open ocean in the MIZ. For instance, wave radiation stress is only briefly mentioned, although these short waves carry more momentum than swell. The results suggest this mechanism could significantly affect polynya opening rates by transferring momentum accumulated over the fetch to the ice edge. Stopa et al. (2018) showed that this effect can be comparable to wind stress in the outer MIZ, and it may similarly influence polynya opening rates (which could even be estimated with a simple back-of-the-envelope calculation assuming a given attenuation rate). It could well be as important a the weakening of the ice, which is much harder to estimate.

We thank the reviewer for this suggestion. We selected the extreme-I_{br} events and have estimated the on-ice wave stress due to the convergence of the wave radiation stress by ice-induced wave attenuation. We did so for two different parameterizations of the wave attenuation rate. We indeed find relatively large wave-induced stresses compared with published estimates for swell in the outer MIZ as short waves are attenuated much faster than swell. Based on an approximation of the wind stress, we find that the wave-induced stress can locally reach approximately ten times the estimated wind stress under the high-attenuation scenario and approximately 1.4 times the wind stress under the low-attenuation scenario. Under both attenuation scenarios, the wave-induced stress decreases to approximately one-third of the estimated wind stress within about 1.7 km of the ice edge. While the wave radiation stress has a relatively small spatial footprint we do expect both processes to be complementary.

We have extended our Discussion with an additional paragraph to highlight the potential relevance of the wave radiation stress, given by:

"The inferred thickening and positive concentration anomalies cannot be explained by wave-induced break-up alone, but indicate redistribution of the ice cover under convergent forcing. In this context, wave radiation stress provides a physically linked momentum source: as locally generated waves attenuate, their momentum is transferred to the ice through convergence of the wave radiation stress, which can drive ice drift, compaction, rafting and ice-edge deformation [28, 29, 31, 32]. Our scaling estimates (Section 4.6) suggest that this wave-induced stress can be comparable to, or locally exceed, the wind stress close to the polynya ice edge, particularly under high-attenuation conditions (Suppl. Fig. 12). The estimated stress is also larger than typical estimates for longer-period swell in the outer MIZ [29], reflecting the stronger momentum convergence associated with the rapid attenuation of short, locally generated waves. However, this same rapid attenuation confines the direct forcing to a relatively narrow band of the order of a kilometer around the polynya edge [32]. We therefore interpret the observed response as an interplay between wave-induced break-up, radiation-stress forcing and wind-driven redistribution: break-up preconditions the ice cover for redistribution by reducing its mechanical coherence and producing smaller, more mobile floes, while wind and wave-induced stresses provide the convergent forcing needed to advect, compact and raft the fragmented ice [28, 33]."

RESPONSE TO REVIEWERS' COMMENTS

Reviewer #1 (Remarks to the Author):

This paper makes a strong argument that waves are a central part of polynya dynamics and that the conventional understanding of polynyas as primary wind-driven overlooks waves as important sources of energy and momentum for the polynya-edge ice. Apart from some minor comments listed below, my major concerns have been addressed. I recommend the paper for publication.

L23: It is unnecessarily restrictive to say Antarctic sea ice (there are also coastal polynyas in the Arctic)

We agree, and have removed 'Antarctic'.

L74: This is a small quibble, but it is certainly possible to measure ocean surface waves in ice impacted areas using deep water moorings. For example, this is done routinely in the Arctic by the Beaufort Gyre Exploration Project moorings (i.e., see Thomson and Rogers 2014, https://doi.org/10.1002/2014GL059983)

We agree and have rephrased this to the following (lines 69-72): "**While upward-looking acoustic Doppler current profilers provide a viable alternative for measuring waves from beneath the surface [14,15], deploying such moored instrumentation in Antarctic coastal polynyas is logistically demanding and resource-intensive.**"

Figure 2: What is the difference between the black and gray scatter points?

Grey markers indicate observations impacted by the instrumental noise floor. We have now updated the caption to reflect this.

L105: Fix typo fst -> fast

We thank the reviewer for noticing, this has now been corrected.

L109: "satisfied throughout the deployments": This does not seem to be strictly true in Figure S1. Perhaps you mean it is satisfied while the buoys are in open water crossing the polynya?

The reviewer is correct, this has now been rephrased.

L145: Might be helpful to mark the noise floor on the spectra in Fig 4 or state its expected magnitude in the caption.

We agree, this is now provided.

L230: The need to daily average the wind forcing is another important assumption/source of uncertainty to note as it smooths the high frequency gusts which would be expected to contribute disproportionately to wave growth.

We agree, this has now been added as follows (lines 227-228): **" … including assumptions about the (daily averaged) wind forcing and …"**

L273: Perhaps better to say "wave forcing" than "wave-induced ice break-up" here - you have shown that the strong wave conditions are associated with sea ice reduction, not that the break up (rather than, for example, wave-induced ice transport) caused the ice reduction

We agree and have altered this to **“wave forcing”**.

L276: “A similar analysis…” Based on the S14 figure caption, it does not appear that the updated conditional averaging approach (using short fetch distances) has been applied for these ice thickness maps (or for S15).

We thank the reviewer for noticing. The approach was correctly applied, but we had not updated the caption. This has now been corrected.

L280: I think Fig S14 is cited out of order (S11 and S12 have not been cited yet, and S13 may not be cited at all)

We have corrected the order of the figures in the Supplementary Information.

L315: “further supporting the interpretation of wave-driven localized deformation and thickening” - Sutherland and Dumont 2018 (10.1175/JPO-D-17-0167.1) is a good reference here

We agree and have added the reference.

L332-334: The estimates of wave radiation stress are a great addition to the paper. However, I am struggling to reconcile the finding that that wave radiation stress impacts are confined to ~1 km from the ice edge with the argument that, from a fracture perspective, “waves may remain dynamically relevant over distances of approximately 10–80 km into the pack” (lines 583-584). I understand that these are different processes and that wave radiation stress convergence depends on the attenuation rate while I_br depends on the wave height, but it still seems like there should be a contradiction here. If waves attenuate rapidly enough to impart a strong wave radiation stress convergence in the first ~1 km of the ice pack, how is it that they remain large enough to break the ice more than ten times that distance into the ice pack? I can imagine that it’s possible for both of these things to be true, but it would be helpful if the text in lines 334-338 (“We therefore interpret…”) were more specific about this interplay.

The distinction arises because wave-induced stress depends on the rate at which wave energy attenuates, whereas break-up potential depends on the wave energy remaining at a given location. Consequently, rapidly attenuating wave components can produce strong momentum transfer close to the ice edge, while more slowly attenuating components produce weaker local stresses but can propagate much farther into the ice while remaining energetic enough for break-up. To clarify this in the manuscript, we have added the following to the discussion at lines 331-336: **“This relatively short forcing length scale does not preclude wave-induced fracture farther into the pack, as the rapid attenuation of shorter-wave components results in strong momentum transfer to the ice near the ice edge, whereas the remaining longer-period and more slowly attenuating waves may still be energetic enough to fracture the ice [30,35,36].”**

L523: I think this may be the incorrect reference for this methodology (also for the reference on line 330)? I think you actually mean this (other) Stopa et al. 2018 paper: https://doi.org/10.1073/pnas.1802011115

We thank the reviewer for noticing, this was indeed incorrect.

Fig S12: In the caption, please explain the thin line width and bold lines. Is this a subset of all polynya events and their mean?

The reviewer is correct, this information has now been added in the caption.

Reviewer #2 (Remarks to the Author):

This is my second review of the manuscript entitled “Inside-outward break-up of sea ice by locally generated waves in the Vincennes Bay Polynya,” submitted to Nature Communications.

As noted in my first review, the study is thorough, the methods are appropriate, and the results support the conclusions. The authors have addressed my comments very well. In particular, Section 2 is now much easier to read, as the purpose of each tool and diagnostic is presented more clearly. The additional work on conditional averaging, together with the discussion of wave radiation stress, also strengthens the authors’ argument that waves contribute to the redistribution of sea ice along the margins of the polynya. I therefore support the publication of this manuscript in Nature Communications.

typo:
L105--> fast ice

We thank the reviewer for noticing. This has been corrected.
